# Orientation‐Driven Large Magnetic Hysteresis of Er(III) Cyclooctatetraenide‐Based Single‐Ion Magnets Adsorbed on Ag(100)

**DOI:** 10.1002/smsc.202400115

**Published:** 2024-04-24

**Authors:** Vladyslav Romankov, Moritz Bernhardt, Martin Heinrich, Diana Vaclavkova, Katie Harriman, Niéli Daffé, Bernard Delley, Maciej Damian Korzyński, Matthias Muntwiler, Christophe Copéret, Muralee Murugesu, Frithjof Nolting, Jan Dreiser

**Affiliations:** ^1^ Paul Scherrer Institute Forschungsstrasse 111 5232 Villigen PSI Switzerland; ^2^ Swiss Nanoscience Institute University of Basel Klingelbergstrasse 82 4056 Basel Switzerland; ^3^ Department of Inorganic Chemistry and Applied Biosciences ETH Zürich Vladimir‐Prelog‐Weg 1‐5/10 8093 Zürich Switzerland; ^4^ Department of Chemistry and Biomolecular Sciences University of Ottawa 150 Louis‐Pasteur Pvt Ottawa ONK1N6N5 Canada; ^5^ Department of Chemical and Physical Sciences University of Toronto Mississauga 3359 Mississauga Road Mississauga ON L5L 1C6 Canada

**Keywords:** magnetism, scanning‐tunneling microscopy, single‐ion magnets, single‐molecule magnets, surfaces, X‐ray absorption, X‐ray photoemission

## Abstract

The molecular self‐assembly and the magnetic properties of two cyclooctatetraenide (COT)‐based single‐ion magnets (SIM) adsorbed on Ag(100) in the sub‐monolayer (ML) range are reported. Our study combines scanning‐tunneling microscopy, X‐ray photoemission spectroscopy and polarized X‐ray absorption spectroscopy to show that Cp*ErCOT (Cp* = 1,2,3,4,5‐pentamethylcyclopentadienide anion) SIMs self‐assemble as alternating compact parallel rows including standing‐up and lying‐down conformations, following the main crystallographic directions of the substrate. Conversely, K[Er(COT)_2_], obtained from subliming the [K(18‐c‐6)][Er(COT)_2_]·2THF salt, forms uniaxially ordered domains with the (COT)^2−^ rings perpendicular to the substrate plane. The polarization‐dependent X‐ray absorption spectra reproduced by the multiX simulations suggest that the strong in‐plane magnetic anisotropy of K[Er(COT)_2_]/Ag(100) and the weak out‐of‐plane anisotropy of Cp*ErCOT/Ag(100) can be attributed to the strikingly different surface ordering of these two complexes. Compared to the bulk phase, surface‐supported K[Er(COT)_2_] exhibits a similarly large hysteresis opening, while the Cp*ErCOT shows a rather small opening. This result reveals that despite structural similarities, the two organometallic SMMs have strongly different magnetic properties when adsorbed on the metal substrate, attributed to the different orientations and the resulting interactions of the ligand rings with the surface.

## Graphical abstract

Molecular single‐ion magnets (SIMs) exhibit a magnetic hysteresis that is known to be strongly influenced by the interaction with the SIM's environment. The present study shows that, surprisingly, two structurally very similar organometallic SIMs adsorbed on a Ag(100) surface can have strikingly different net magnetic anisotropies and hysteretic behaviors because of different self‐assembly properties and adsorption conformations.

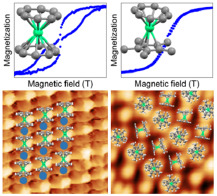

## 1 Introduction

Single‐molecule magnets (SMMs) exhibit magnetic hysteresis without the presence of magnetic long‐range order. This group of materials includes exchange‐coupled molecular clusters with several metal ions and molecular complexes based on only one ion.^[^ ^1^, ^2^, ^3^, ^4^, ^5^, ^6^, ^7^, ^8^ ^]^ The latter are often denoted as molecular single‐ion magnets (SIM). In this context, lanthanide ions are appealing due to their large unquenched orbital magnetic moments, high spin ground states, and the possibility of tuning the magnetic anisotropy via proper ligand environment.^[^ ^9^, ^10^ ^]^ Sandwich‐type complexes based on phthalocyanine, cyclopentadienide (Cp), and/or cyclooctatetraenide (COT) ligands, as well as their derivatives, have been shown to induce large magnetic anisotropy on lanthanide ions such as Tb^3+^, Dy^3+^, and Er^3+^.^[^ ^5^, ^11^, ^12^, ^13^, ^14^, ^15^, ^16^ ^]^ In some cases, it has been shown that the complexes preserve the hysteresis opening even above liquid nitrogen temperature.^[^ ^17^ ^]^ This makes these coordination compounds attractive for ultrahigh‐density memory applications. In turn, for any device applications, the two‐dimensional ordering of the SIMs on planar surfaces is of vital importance to access the magnetic information by local probes, for example, using a scanning probe microscopy tip.^[^ ^18^ ^]^ The molecule–substrate interaction is an important factor affecting most often negatively the hysteresis loop openings upon surface adsorption of SMMs and SIMs.^[^ ^18^, ^19^, ^20^, ^21^, ^22^, ^23^, ^24^ ^]^ In other cases, strong molecule–surface interaction promotes charge transfer that can completely alter the magnetic properties of molecules on metals in the ML range.^[^ ^25^ ^]^ A few exceptions rely on the use of long chemical linkers or stiff buffer layers to quench vibrational relaxation pathways, while also eliminating the hybridization with the metallic surface.^[^ ^26^, ^27^, ^28^ ^]^

**Figure 1:**
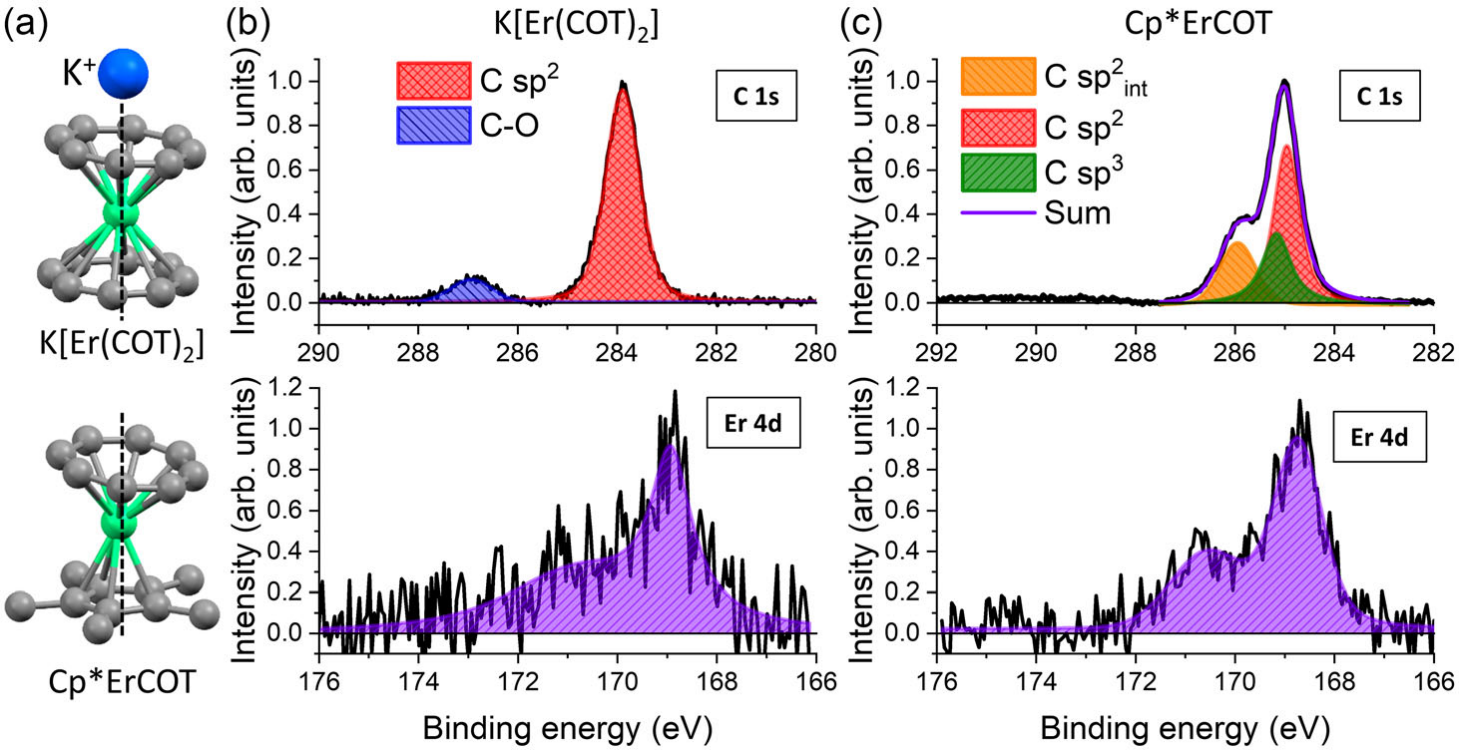
a) Molecular structures of K[Er(COT)_2_] (top) and Cp*ErCOT (bottom). Color code: green: Er^3+^; gray: C; blue: K. Hydrogen atoms are omitted for simplicity. C 1s and Er 4d normalized core level spectra of b) K[Er(COT)_2_](≈0.5 ML)/Ag(100) and c) Cp*ErCOT(≈1 ML)/Ag(100).

Here we study two surface‐adsorbed mononuclear Er^3+^ sandwich‐type SIMs, the neutral Cp*ErCOT (with (Cp*)^−^ the 1,2,3,4,5‐pentamethylcyclopentadienide anion and (COT)^2−^ the cyclooctatetraenide dianion)^[^ ^29^ ^]^ and the ionic K[Er(COT)_2_], with the structures shown in **Figure** **1**a. K[Er(COT)_2_] is obtained by sublimation of [K(18‐c‐6)][Er(COT)_2_]·2THF (where THF is tetrahydrofuran and “18‐c‐6” denotes the 18‐crown‐6 ether). Both Cp*ErCOT and [K(18‐c‐6)][Er(COT)_2_]·2THF show strong magnetic anisotropy with the easy axis aligned perpendicular to the almost parallel ligand rings,^[^ ^30^, ^31^, ^32^, ^33^ ^]^ similar to other COT‐based (and their functionalized analogues) SMMs.^[^ ^34^, ^35^, ^36^, ^37^, ^38^ ^]^

We focus on comparing the self‐assembly and the magnetic properties of these SIMs in the sub‐ML to the few‐layer range adsorbed on the (100) surface of an Ag single crystal. A strikingly different ordering and self‐assembly of the two compounds is unveiled by scanning‐tunneling microscopy (STM) and X‐ray linear dichroism (XLD). Point‐charge‐based simulations as implemented in the multiX code^[^ ^39^ ^]^ are compared to polarized X‐ray absorption spectra to understand the net magnetic anisotropy in connection with the ordering of the SIMs. Finally, X‐ray magnetic circular dichroism (XMCD) is used to detect the element‐specific magnetic moments and the magnetic hysteresis loops.

## 2 Results and Discussion

### 2.1 Chemical Characterization

Samples of surface‐adsorbed K[Er(COT)_2_] and Cp*ErCOT were obtained by sublimation of polycrystalline powder onto a freshly prepared Ag(100) surface in ultrahigh vacuum. An ML corresponds to a densely packed single layer of molecules. Normalized X‐ray photoelectron spectroscopy (XPS) results of K[Er(COT)_2_](≈0.5 ML)/Ag(100) and Cp*ErCOT(≈1 ML)/Ag(100) are reported in Figure 1b,c, respectively. The K[Er(COT)_2_]/Ag(100) has the main peak of the C 1s core level at a binding energy of 283.9 eV (full width at half maximum (FWHM) 0.8 eV) and a smaller peak at 286.9 eV (FWHM 1.1 eV).

We ascribe the former peak to the *sp* ^2^‐hybridized carbon atoms of (COT)^2−^, while the small feature at higher binding energy is consistent with the presence of oxygen‐bound carbon. Since the [K(18‐c‐6)][Er(COT)_2_]·2THF salt was used as source material, it is not surprising to find a small fraction of tetrahydrofuran (THF) and (18‐c‐6) crown ether molecules deposited on the surface as well.^[^ ^40^, ^41^ ^]^ However, as shown by XPS spectra recorded at different crucible temperatures, the volatile crown ether and THF are almost completely removed from the source material below the sublimation temperature of K[Er(COT)_2_] (see Supporting Information). The lower binding energy of the C 1s levels with respect to reported charge neutral metallocenes^[^ ^42^ ^]^ is attributed to the charge separation and the resulting coverage dependent electric dipole formation that the complex undergoes upon direct interaction with the substrate, as discussed further below and in the Supporting Information. Note that because of this the C 1s binding energies cannot be directly compared with the ones of Cp*ErCOT and literature references. Nevertheless, the atomic ratio of Er, K and *sp* ^2^ C corresponds to the stoichiometry expected for K[Er(COT)_2_] complexes (see discussion in the Supporting Information).

The analysis of the C 1s core level spectrum of Cp*ErCOT/Ag(100) as shown in Figure 1c yields three contributions: a peak at 284.9 eV (FWHM 0.6 eV), attributed to the *sp* ^2^‐hybridized carbon atoms of the aromatic rings^[^ ^42^, ^43^, ^44^ ^]^; a peak at 285.2 eV (FWHM 0.7 eV), attributed to the *sp* ^3^ carbon atoms of the methyl groups in the Cp* ligand^[^ ^45^ ^]^; and a peak at 285.9 eV (FWHM 0.8 eV), attributed to the *sp* ^2^‐hybridized carbon atoms of the COT ligands interacting with the metal substrate, as discussed further below. The Er 4 d core levels appear at characteristic energies for the trivalent Er^3+^ ion,^[^ ^46^ ^]^ with the main peak of the K[Er(COT)_2_] centered at 168.9 eV, while the one of Cp*ErCOT SIMs lies at 168.7 eV. In order to obtain the stoichiometry from the XPS scans, the energy‐dependent cross‐sections and asymmetry parameters were taken from tables reported in the literature.^[^ ^47^ ^]^ The extracted C:Er atomic ratio for the Cp*ErCOT SIMs is 21:1, while the K[Er(COT)_2_] shows a C:Er ratio of 16:1, a K:Er ratio of 0.8:1 and O:Er ratio is 2.5:1. Within the error bar of ≈30%, which is mainly due to the weak Er 4 d signal used to estimate the ratios, these values are in excellent agreement with the atomic ratios expected from the molecular structures, pointing to the integrity of the complexes on the surface.

### 2.2 Surface Ordering

**Figure 2:**
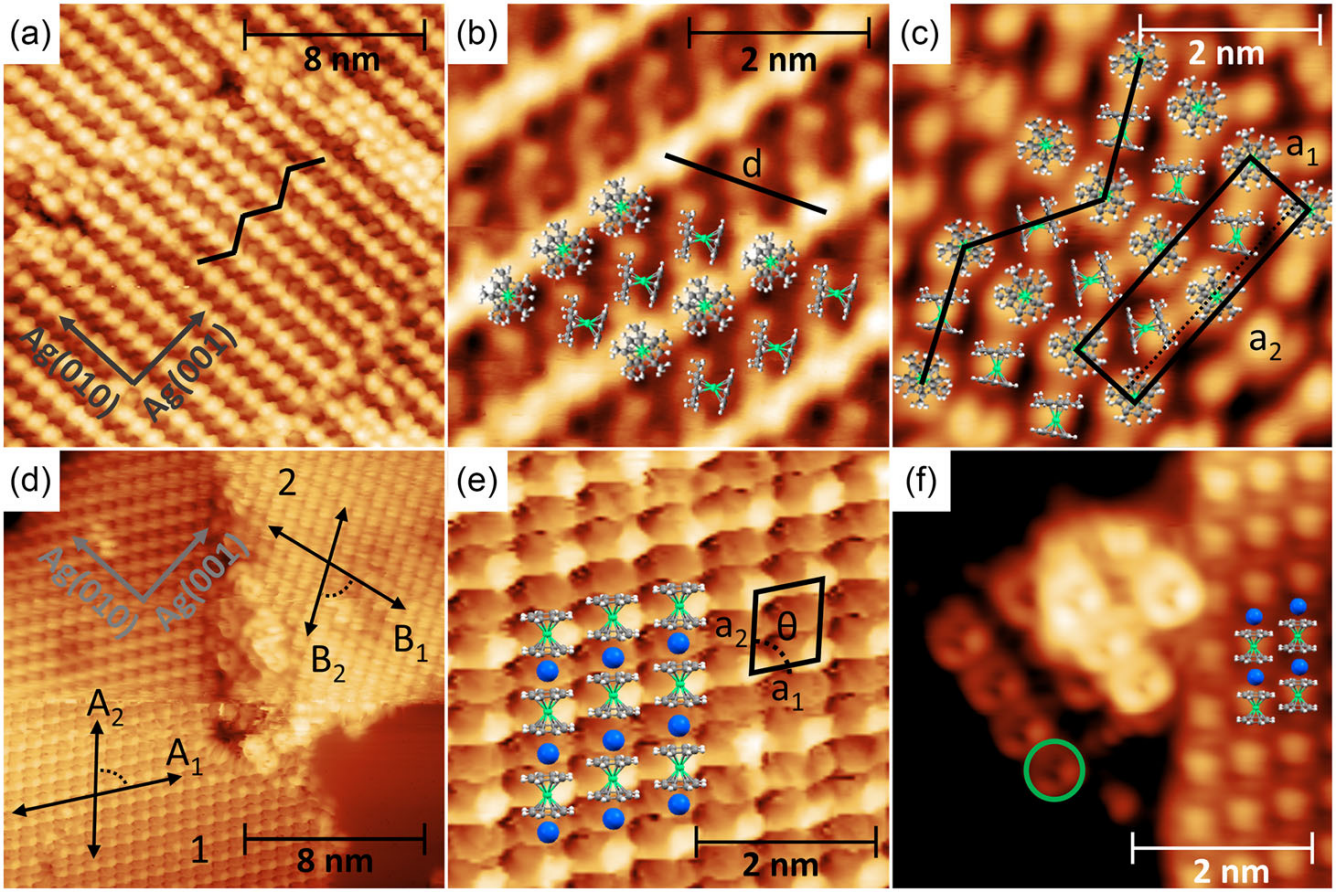
Constant current STM images at 4.5 K of a–c) Cp*ErCOT and d–f) K[Er(COT)_2_] adsorbed on Ag(100). Imaging conditions: (a) 0.8 V; 200 pA; (b) 0.25 V; 50 pA; (c) 2 V; 500 pA; (d) −2 V; 50 pA; (e) zoom into (d); (f) −2 V; 50 pA. Molecular overlays, the herringbone structure, the molecular orientation indicated by the black arrows, and the unit cells are shown, as explained in the main text. Color code: green: Er; gray: C; blue: K; white: H.

The STM images of Cp*ErCOT(≈1 ML)/Ag(100) are presented in **Figure** **2**a–c. Alternating rows of brighter and darker spots can be identified in the 20 × 20 nm^2^ image in Figure 2a. The rows are oriented along the [010] and [001] crystallographic directions of the Ag substrate and they form domains of a few tens of nanometers, that cover densely the surface. The average distance between two rows of bright spots amounts to 1.48 ± 0.04 nm, while the distance between two height maxima within the same row is 0.84 ± 0.05 nm. We ascribe the different spots to two different adsorption conformations, with the orientation of the complexes identified by their main rotational axis (dashed lines in Figure 1a). In Figure 2a, the distances between the spots are consistent with alternating rows of standing‐up and lying‐down complexes on the surface, as detailed further below. In some parts of the islands, the ordering of the complexes follows a herringbone structure as shown by the black pattern in Figure 2a. This can be explained by the orientation of the main molecular axes of the lying‐down complexes being rotated at an angle in the surface plane, (see overlay in Figure 2c). The distance between two standing‐up complexes in this rotated direction amounts to *d* = 1.54 ± 0.04 nm, perfectly matching the distance of 1.55 nm (at 10 K) that the complexes have along the [‐101] direction in the crystal structure of pure Cp*ErCOT, with the same standing‐up and lying‐down geometry.^[^ ^29^ ^]^ However, the surface ordering does not correspond to the one in any of the planes present in the crystal structure of the parent compound.

The bright spots in Figure 2b are ascribed to the up‐right complexes, with the (Cp*)^−^ ring being on the top, resembling in size and shape the one reported for [Cp*Ru]^+^ ions on graphene.^[^ ^48^ ^]^ The bright lobes of the lying‐down complexes are attributed to the methyl groups, while the darker areas are attributed to the (COT)^−^ rings, poorly conducting in the direction perpendicular to the π‐bonds.^[^ ^49^, ^50^ ^]^ From Figure 2b,c, the molecular density is estimated to approximately 1.7 molecules nm^−^^2^.

Figure 2c shows a zoom into part of the area reported in Figure 2a, sampled at a bias voltage of *V*_b_ = 2 V and a current setpoint of *I*_s_ = 500 pA. In this condition, the apparent heights of the two rows become comparable, and the lying‐down complexes are seen as single bright spots, possibly due to an enhanced conduction through the Er^3+^ ion at these imaging conditions. The overlay presented in the figure shows that the herringbone structure mentioned previously probably originates from the alternating orientation of the complexes in the lying‐down rows. The superstructure formed by the complex can be described by the unit cell reported in Figure 2c, with *a*_1_ × *a*_2_ = 0.84 × 2.96 nm^2^ and the vectors oriented along the [010] and [001] directions of the substrate. The black dotted line shows the lateral shift of 0.15 ± 0.02 nm of every second row. Note that defect rows are present in the form of three consecutive rows of standing‐up complexes as seen, e.g., on the upper right corner of Figure 2a. Furthermore, STM images showing the coverage, and the terrace steps are reported in the Supporting Information.

The STM images of K[Er(COT)_2_](0.5 ML)/Ag(100) are reported in Figure 2d–f. Figure 2d shows two different domains of highly ordered complexes, labeled as 1 and 2, with the orientation of the rows indicated by black arrows. In both domains, the directions labeled *A*_1_, *A*_2_, and *B*_1_, and *B*_2_ forms an angle of 75°. With respect to the Ag(001), the direction labeled *A*_1_ is rotated clockwise by ≈35°, while the one labeled *B*_1_ is rotated clockwise by ≈80°. The complexes of both domains form highly oriented compact rows, as seen in the zoom into domain 1, reported in Figure 2e. The latter figure shows a repeating pattern of ovals with an approximate size of 0.44 nm × 0.74 nm exhibiting a rhombic assembly, with a brighter feature on every second oval along the *A*_2_ direction of the domain (*B*_2_ on domain 2). The size of the ovals is consistent with the one of the [Er(COT)_2_]^−^ anions lying down on the substrate (≈0.4 × 0.6 nm),^[^ ^29^ ^]^ with the molecular axis parallel to the surface plane. The modulation of the local density of states is likely given by the alternation of K^+^ and Er^3+^ ions, separated by standing COT^2−^ rings, as shown by the overlay in Figure 2e. This stacking of Er – COT – K – COT forms uniaxial rows of densely packed K[Er(COT)_2_] complexes. The areal molecular density is estimated to be 1.6 molecules nm−^2^. The unit cells of both domains (1 and 2) form a rhombic shape with *a*_1_ = 0.74 ± 0.05 nm, *a*_2_ = 0.88 ± 0.04 nm, and *θ* = 75°, as shown in Figure 2e (in domain 2 the unit cell is mirrored with respect to *a*_2_). However, the vectors of the unit cells are not aligned along any of the principal crystallographic directions of the substrate. Finally, in some areas, small agglomerates of other molecules are found as shown in Figure 2f. The round‐shaped molecules have a diameter of 0.90 ± 0.05 nm as highlighted by a green circle, and the size is consistent with the (18‐c‐6) crown ether of the source material (see also Methods).

### 2.3 Orientation of Self‐Assembled Complexes

Linearly polarized X‐rays probe empty orbitals of the absorbing atom oriented along the oscillating electric field of the X‐rays. Thus, a symmetry breaking of the system gives rise to a difference between absorption spectra measured with different linear X‐ray polarizations, the so‐called XLD. Similarly, circularly polarized X‐rays can probe in an element‐specific way the projection of the sample magnetization onto the beam direction. By taking the difference of two absorption spectra with opposite circular polarization, the XMCD is obtained.^[^ ^51^ ^]^

The XLD and XMCD measurements are complemented by simulations based on a point‐charge model implemented in the multiX software.^[^ ^39^ ^]^ The point charges were put at the D_8h_‐symmetrized positions of the carbon atoms as explained in the Supporting Information. In particular, the detailed shape of the triple‐featured Er^3+^ M_5_‐edge, which depends on the orientation of the X‐ray polarization and the incidence angle with respect to the main axis of the SIMs, is reproduced in the calculations.

**Figure 3:**
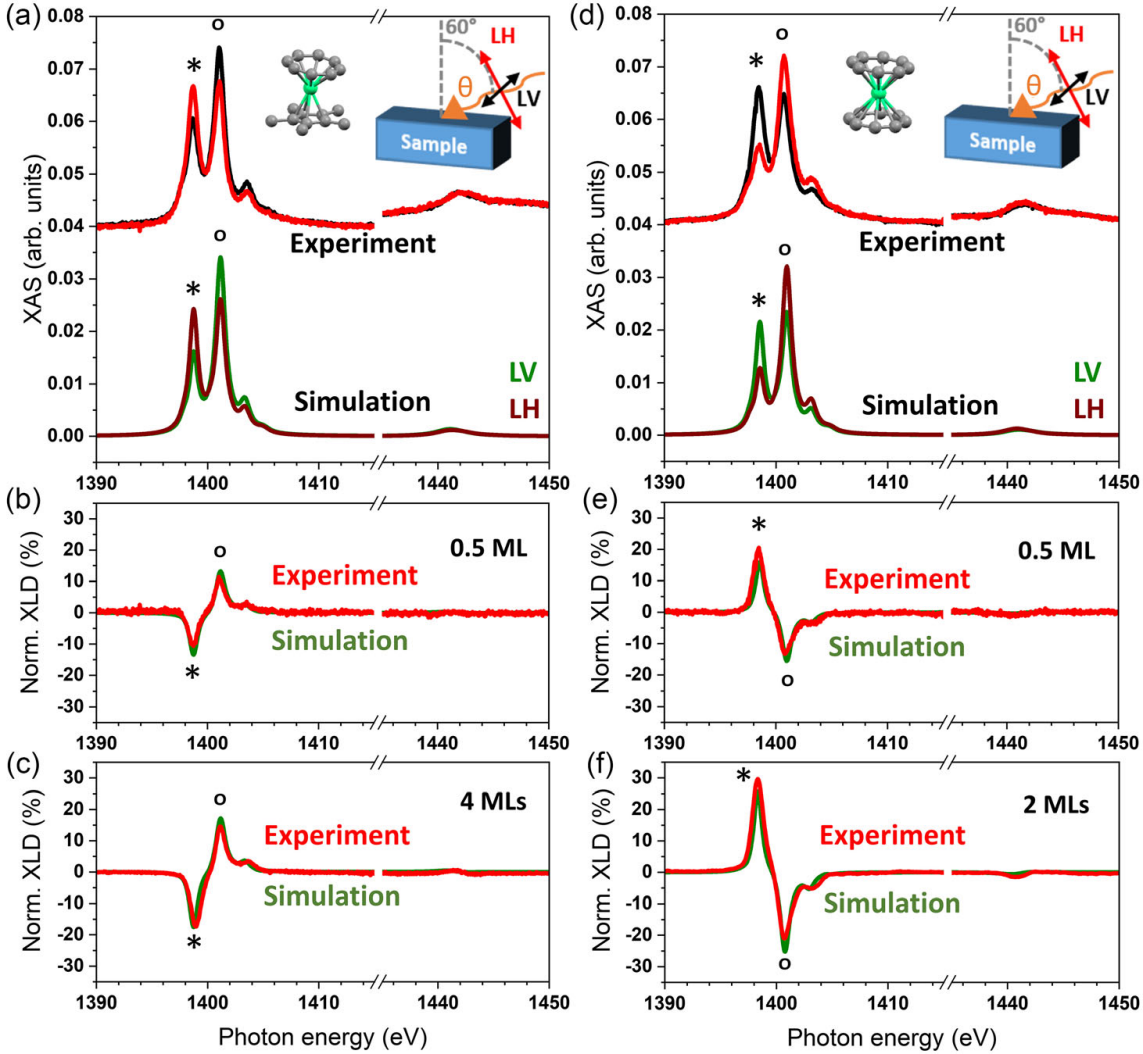
Linearly polarized XAS recorded at the Er M_4,5_ edges at 3 K and 50 mT compared to multiX‐simulated spectra of a) Cp*ErCOT(0.5 ML)/Ag(100) and d) K[Er(COT)_2_](0.5 ML)/Ag(100). The insets show the molecular structures (H atoms omitted) and the experimental geometry of the grazing‐angle measurements, along with the polarization and the beam direction. Panels b,c), and e,f) show the experimental and simulated XLD spectra of different samples as indicated in the plots. The symbols * and ° identify the main features as described in the main text.

In **Figure** **3**, X‐ray absorption spectroscopy (XAS) results obtained using linearly polarized X‐rays and XLD spectra are reported. Cp*ErCOT(0.5 ML)/Ag(100) (see Figure 3a) shows a similar absorption intensity for the two peaks identified as * (1398.8 eV) and ° (1401.1 eV) in the out‐of‐plane direction of the substrate (LH, red curve), whereas the in‐plane polarized absorption (LV, black curve) is more intense in the ° peak. The simulation shows that the intensities of the * and ° peaks are given by the orientation of the complex with respect to the incoming photons and their polarization (see Supporting Information). Based on the results of the STM images, we have reproduced the spectra by using a model with an equal amount of complexes in the standing‐up and lying‐down configurations. In the case of the lying‐down configuration, the main molecular axis was taken parallel to the substrate plane with uniform disorder over all azimuthal angles. Manual adjustment of the point‐charge values of the C atoms (*q*_C_) yielded the best agreement between simulated and experimental spectra when fixing the value to *q*_C_ = 0.25 *e*. Figure 3b shows the comparison of the experimental and simulated XLD, with the intensities reported in **Table** **1**. The good agreement of the XLD shape points to the fact that the negative sign of * and the positive sign of ° are characteristic of a mixed standing‐up and lying‐down configuration of the complexes on the surface. The excellent match of the simulated XLD spectra with the experimental ones suggests that both adsorption configurations occur with a ratio of 1:1.

**Table 1:** XLD intensities of * (≈1398.5 eV) and ° (≈1401 eV) peaks of experimental and simulated spectra (multiX) of Cp*ErCOT/Ag(100) and K[Er(COT)_2_]/Ag(100), as reported in Figure 3 and S7 of the Supporting Information

	XLD * [%]		XLD ° [%]	
	Experiment	multiX	Experiment	multiX
Cp*ErCOT (0.5 ML)	−11 ± 2	−13.4	11 ± 2	13.1
Cp*ErCOT (4 MLs)	−17 ± 3.5	−17.5	15 ± 3	17.2
K[Er(COT)_2_] (0.5 ML)	21 ± 4	15.8	−13 ± 3	−15.5
K[Er(COT)_2_] (2 MLs)	30 ± 6	25.8	−21 ± 4	−25.2

Multilayer samples show a similar shape of the XLD as shown in Figure 3c. While the XAS of the 4 ML sample reported in the (Figure S6, Supporting Information) is very similar compared to the sub‐ML sample, the spectra are better reproduced by assuming that 55% of the complex are in the standing‐up configuration, in good agreement with the experimental results presented in Figure 3 and Table 1.

On the contrary, K[Er(COT)_2_](0.5 ML)/Ag(100) shows a similar absorption intensity for both * (1398.3 eV) and ° (1400.6 eV) peaks in the substrate plane (black curve), as reported in Figure 3d. There is a strong absorption asymmetry (red curve) in the out‐of‐plane direction, with the main contribution coming from the ° peak. This result agrees with the adsorption almost purely in the lying‐down configuration, as revealed by the simulations, which reproduce very well the experimental XAS and XLD reported in Figure 3d,e. Note that a good fit of the XLD in Figure 3e and of the XMCD spectra shown further below can only be obtained by taking into account a fraction of 13% of the complexes adsorbed in the standing‐up geometry. This can be rationalized by assuming that in the sub‐ML coverage not all complexes are engaged in the self‐assembled islands. In analogy to other complexes containing similar organic macrocycles, the isolated molecules prefer the standing‐up geometry with the rings parallel to the metal surface plane.^[^ ^44^ ^]^ The 2 MLs K[Er(COT)_2_]/Ag(100) sample exhibits the same shape of the XLD as visible in Figure 3f (see also Figure S6, Supporting Information), with an overall larger intensity (see Table 1). Here, no standing‐up complexes were assumed in the simulations.

**Figure 4:**
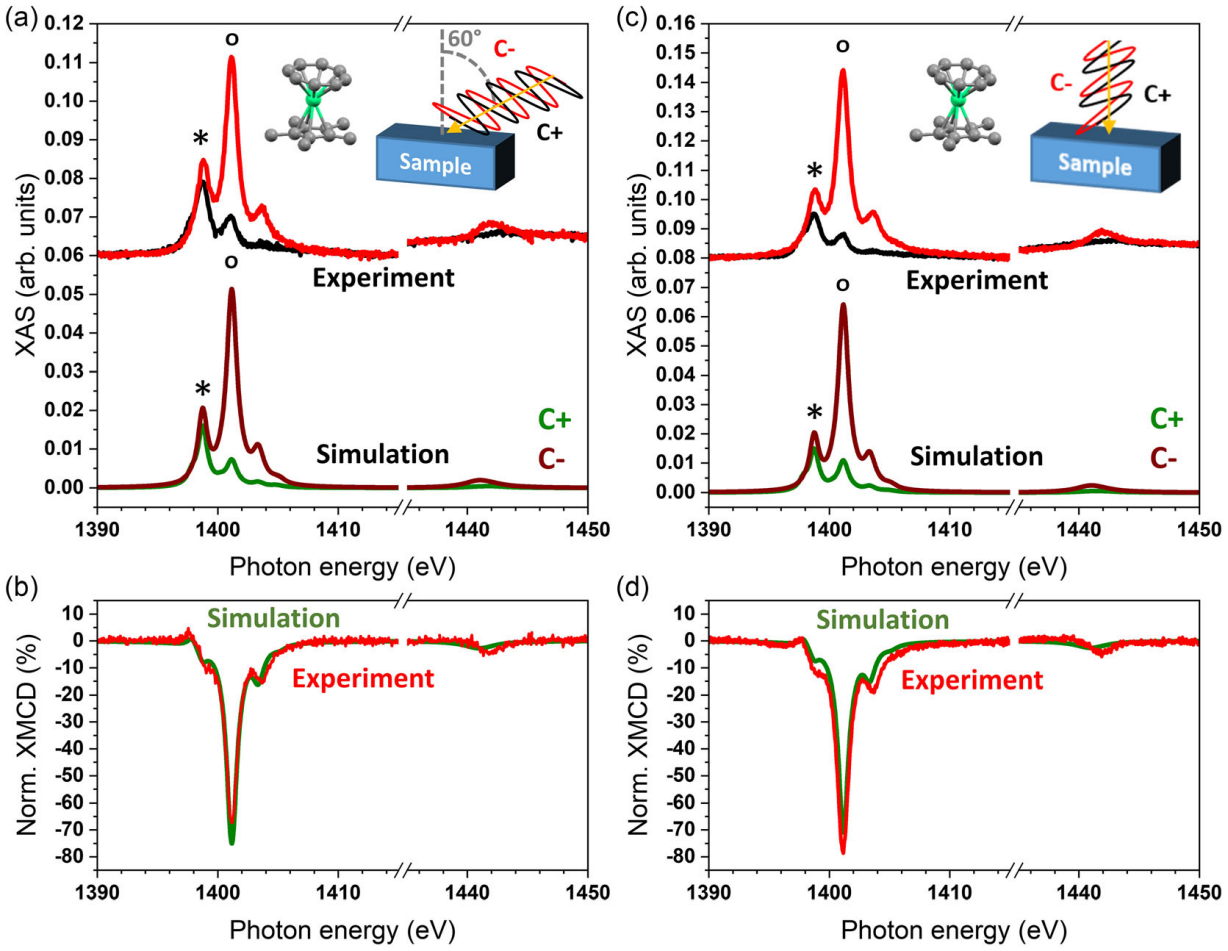
Circularly polarized X‐ray absorption and XMCD spectra measured at the Er M_4,5_‐edges at 3 K and 6.8 T, along with multiX‐simulated spectra of Cp*ErCOT(0.5 ML)/Ag(100). a,b) Grazing (60°) incidence and c,d) normal (0°) incidence as shown in the insets.

The strong easy‐axis type magnetic anisotropy of the individual complexes can be used to rationalize the surface ordering by directly comparing XMCD intensities and thus the magnetic moments in normal and grazing incidence. **Figure** **4** shows circularly polarized XAS and XMCD of Cp*ErCOT(0.5 MLs)/Ag(100). By using the previously explained multiX model with the equal standing‐up versus lying‐down ratio, the simulated XAS reproduces well the experimental results in the figure. The XMCD spectra in Figure 4b,d are also well reproduced, with the intensities of the strongest (negative) peaks at 1401.2 eV reported in **Table** **2**. For the multilayer, the XMCD spectra of both the normal and grazing incidence become comparable in intensity. The simulations based on the 55:45 standing‐up vs. lying‐down molecular arrangement described in the XLD section perfectly fit the results in Table 2.

**Table 2:** Experimental and simulated XMCD intensities of Cp*ErCOT and K[Er(COT)_2_] spectra reported in Figure 4, 5, S7, and S8 of the Supporting Information

	XMCD normal [%]		XMCD grazing [%]	
	Experiment	multiX	Experiment	multiX
Cp*ErCOT (0.5 ML)	87 ± 9	71.0	65 ± 7	75.0
Cp*ErCOT (4 MLs)	74 ± 7	74.6	75 ± 8	75.6
K[Er(COT)_2_] (0.5 ML)	39 ± 4	34.7	75 ± 8	69.9
K[Er(COT)_2_] (2 MLs)	16 ± 2	16.4	73 ± 7	68.0

**Figure 5:**
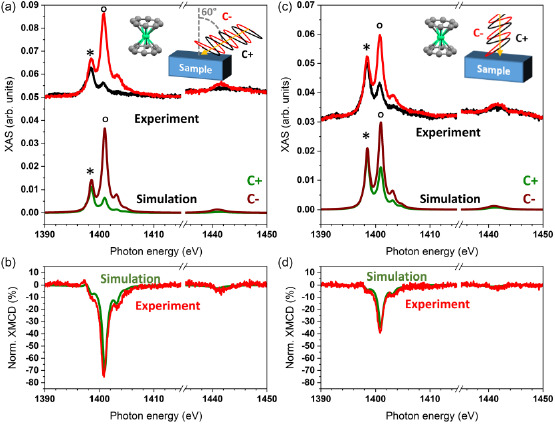
Circularly polarized XAS and XMCD recorded at the Er M_4,5_‐edges at 3 K and 6.8 T, along with multiX‐simulated spectra of K[Er(COT)_2_](0.5 ML)/Ag(100). a,b) Grazing (60°) and c,d) normal incidence (0°) as shown in the insets.

Opposite to the Cp*ErCOT SMMs, the circularly polarized spectra of K[Er(COT)_2_](0.5 ML)/Ag(100) show a strong difference between grazing and normal incidence of the X‐rays, as presented in **Figure** **5**a,c. While the ° peak of the C^−^ polarization is the dominant one in both cases, it is far less intense in normal incidence. As introduced previously, the best‐fit simulations are based on the model with 87% of the complexes self‐assembled in the lying‐down configuration, isotropically oriented over the azimuthal angles, and 13% in the standing‐up geometry. There is excellent agreement between the simulations and the experimental spectra as manifested in the spectra shown in Figure 5, as well as in the peak heights collected in Table 2.

Increasing the thickness of the sample to 2 MLs does not affect much the shape of the XAS compared to the sub‐ML (see Figure S7, Supporting Information), but the XMCD intensity in normal incidence drastically decreases. This is ascribed to the absence of standing‐up complexes in the multilayer sample.

The sum rule analysis^[^ ^52^, ^53^ ^]^ of the XMCD spectra was performed to obtain the net spin and orbital magnetic moments of the erbium ion. The results are summarized in Table S2, Supporting Information, and discussed in the Supporting Information. The Cp*ErCOT(0.5 ML)/Ag(100) shows a total magnetic moment of 5 ± 1 μ_B_ in normal incidence, about half of the expected value for the pristine complex, attributed to the mixed ordering and azimuthal disorder of the complex. For K[Er(COT)_2_], the total magnetic moment of 4.5 ± 0.8 μ_B_ in grazing against the 1.9 ± 0.8 μ_B_ in normal incidence fits the easy‐plane type anisotropy of the net magnetization resulting from the in‐plane, lying‐down, ordering. The XAS and XMCD obtained on the powder sample of the starting material [K(18‐c‐6)][Er(COT)_2_]·2THF (cf. Figure S9, Supporting Information) exhibit the same shapes as observed on the surface‐adsorbed complexes. Furthermore, the corresponding sum rule analysis yields a total magnetic moment of 4.3 ± 0.5 μ_B_ consistent with the randomly oriented anisotropy axes of the complexes in the powder as discussed in the Supporting Information.

### 2.4 Magnetic Hysteresis Loops

**Figure 6:**
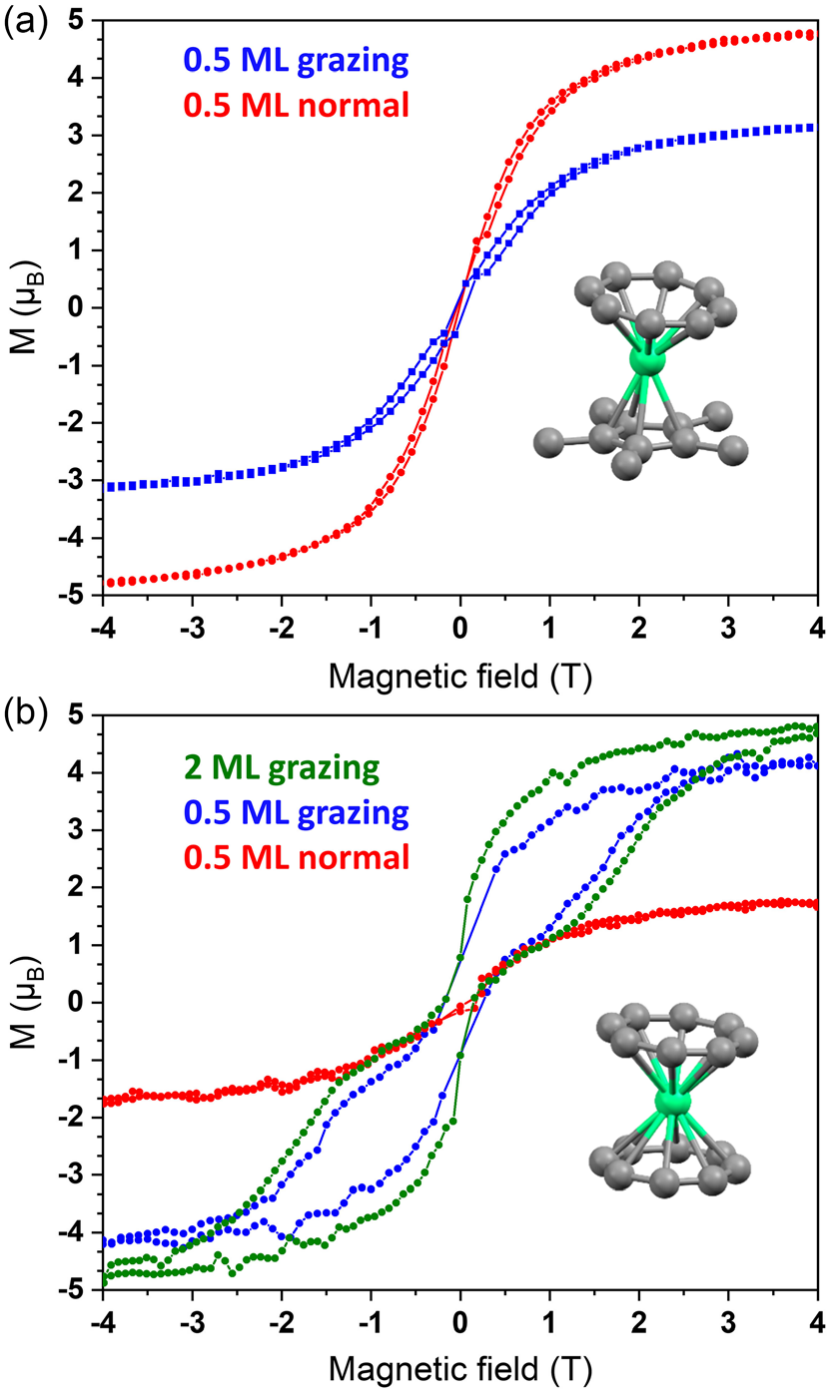
XMCD‐detected magnetic hysteresis loops recorded at 3 K of a) K[Er(COT)_2_] and b) Cp*ErCOT adsorbed on Ag(100), at a rate of 2 T min^−1^. Normal stands for out‐of‐plane direction, while grazing (60°) is mostly in‐plane. Magnetic moment values are obtained from sum rule analysis. Lines connecting experimental points are guides to the eyes.

**Figure** **6**a displays the magnetic hysteresis of Cp*ErCOT(0.5 ML)/Ag(100), measured at a ramping speed of 2 T min^−1^. Only a small hysteresis opening is visible for both incidence angles. The hysteresis loops of the 4 ML samples (Figure S10, Supporting Information) are of the same shape as the ones of the sub‐ML sample. However, the saturation value is different (see Table S2, Supporting Information). Since bulk Cp*ErCOT shows a substantial butterfly‐like hysteresis opening at 3 K,^[^ ^54^ ^]^ the reduction of the hysteresis opening of the surface adsorbed Cp*ErCOT can be attributed to the interaction with the substrate, as discussed later.

The magnetic hysteresis of the K[Er(COT)_2_](0.5 ML)/Ag(100) sample shown in Figure 6b exhibits a large butterfly‐like opening between ±3.5 T in grazing incidence, while the loop is essentially closed in normal incidence. This is in agreement with the ordering of the net easy axis of the magnetization of the complexes parallel to the substrate plane. The different values of the magnetic moments in the two directions are another clear evidence of the net magnetic anisotropy of the sample due to the surface ordering of the complexes, as discussed in the previous section. The hysteresis of the multilayer sample exhibits a very similar, but larger opening in grazing incidence of the X‐rays (see also Figure S11, Supporting Information). For both coverages, the coercive field was estimated to be *B*_coerc_ = 0.15 ± 0.07 T, while the remnant magnetization as *M*_rem_ = 0.84 ± 0.06 μ_B_. The fact that the hysteresis loop of the 2 MLs sample shows a larger loop opening indicates that the multilayer sample has greater magnetic stability.

### 2.5 Discussion

Cp*ErCOT and K[Er(COT)_2_] in the sub‐ML to ML range exhibit completely different self‐assemblies on Ag(100). Cp*ErCOT forms domains of alternating rows of standing‐up and lying‐down complexes as shown by STM and XAS. A similar ordering was observed for metallocenes on metal substrates, where such a configuration is dictated by the T‐shaped van der Waals interaction between the Cp ligands of neighboring molecules.^[^ ^49^, ^55^ ^]^ The presence of lying‐down nickelocene was shown to be necessary to minimize the adsorption energy of the molecules on metals,^[^ ^55^ ^]^ and it is likely the case also for the Cp*ErCOT studied here. The main difference compared to the metallocenes is that the lying‐down Cp*ErCOT complex connects “diagonally” two standing‐up complexes of neighboring rows rather than perpendicularly to the standing‐up rows of complexes, reproducing the ordering formed along the [‐101] direction in the Cp*ErCOT unit cell. Also, the orientation of lying‐down rows changes between consecutive rows, forming a herringbone‐like pattern, as shown in Figure 2a,c. The herringbone structure is found also in the compact configuration of metallocenes,^[^ ^49^ ^]^ caused by the lateral shift of 0.15 nm by standing‐up complexes belonging to every second row, which is also observed in Cp*ErCOT/Ag(100). This likely happens so the complexes can accommodate in the energetically favorable adsorption sites on the surface, while still being influenced by the intermolecular interaction.

On the other hand, heteroleptic sandwich complexes can deviate from this ordering, as recently reported for CpTiCOT/Au(111).^[^ ^44^ ^]^ In that case, the compound forms a complex unit cell of mixed standing‐up and lying‐down complexes in a 1:3 ratio, due to stronger intermolecular interactions. In the present case, STM, XLD, and multiX simulations suggest that equal amounts of the two Cp*ErCOT conformations are present. Although the model used is very simple and based on the highly symmetric D_8h_ symmetry of the point charges for both compounds, it can reproduce the experimental XLD and XMCD spectra with great fidelity. Most importantly, it points out that the reversed sign of the XLD shapes of the two complexes is given by their different orientations. In fact, K[Er(COT)_2_] forms domains of highly oriented rows with lying‐down complexes and with the main molecular axis oriented along the same direction. Such a conformation suggests that the [Er(COT)_2_]^−^ anions are conjugated through the intercalation of K^+^ ions, in a similar manner as reported for the K(THF)_4_[Er_2_COT_4_] tetralayer.^[^ ^34^ ^]^ Indeed, the STM‐measured distance between two K[Er(COT)_2_] complexes is *a*_2_ =  0.88 ± 0.04 nm (cf. Figure 2f), which coincides with the Er‐Er distance in the K(THF)_4_[Er_2_COT_4_] cluster. Altogether, this suggests that K[Er(COT)_2_] forms rows of alternating Er^3+^ and K^+^ ions spaced by standing COT^2−^ rings. A similar linear wire‐like configuration of COT‐based single‐chain magnets on surfaces was reported for EuCOT/Graphene/Ir.^[^ ^50^, ^56^ ^]^ For neutral π‐conjugated systems, a preferential ordering with the rings parallel to the surface plane or driven by the T‐shaped interaction would be expected as discussed previously. In contrast, in the case of K[Er(COT)_2_], the charge balance *via* intercalation of a potassium ion between two complexes is the main mechanism driving the self‐assembly.

The vertical position of the K^+^ ions in the first layer is likely influenced by their charged nature: They adsorb closer to the metal surface, instead of being centered between two (COT)^2−^ rings, as in the reported tetralayer.^[^ ^34^ ^]^ This creates a vertical charge separation with a positive layer on the surface, balanced by the metal surface, and a negatively charged layer of [Er(COT)_2_]^−^ anions. The charge separation in the sub‐ML case forms an electric dipole that acts as a built‐in potential, shifting the positions of the XPS peaks, as reported for other dipolar materials.^[^ ^57^, ^58^ ^]^ By increasing the thickness of the sample, the influence of the surface on the potassium cations becomes less important and more axially aligned complexes are formed. This effect explains the rather low C 1s binding energy of 283.9 eV of the sub‐ML K[Er(COT)_2_] and the core level shifts reported in the Supporting Information, although the contribution of surface screening cannot be excluded. A depiction of the adsorption conformations of the studied complexes is shown in Figure S3, Supporting Information.

We conclude that Cp*ErCOT experiences a stronger interaction with the surface based on three observations. 1) The presence of two different *sp* ^2^ C 1s signals at binding energies of 284.9 and 285.9 eV, representing the noninteracting carbon atoms of the aromatic rings and the ones interacting with the metal substrate (cf. Figure 1): Considering a standing‐up and a lying‐down complex, out of 36 carbon atoms 8 are hybridized with the metal surface because the COT ring of the standing‐up complex has its π‐electron cloud in the metal plane; 18 *sp* ^2^ carbon atoms, the inner ones of the Cp* ligand and the remaining COT of the lying‐down species, contribute to the peak at lower binding energy; the 10 *sp* ^3^ carbons, which are part of the methyl groups of the Cp* ligands give rise to an intermediate peak at 285.2 eV, typical for *sp* ^3^ carbon.^[^ ^45^ ^]^ Consistently, the C 1s peak areas shown in Figure 1c exhibit an 8:10:18 ratio. This scenario is very similar to the interpretation in ref. [45], in which two different C 1s signals at similar binding energies as in the present text were observed (with the exclusion of the *sp* ^3^ peak). 2) The substrate templating effect: The unit cell vectors of the molecular Cp*ErCOT overlayer coincide with the main crystallographic directions of the substrate. 3) The shrinkage of the hysteresis loops of the surface‐adsorbed complexes compared to the bulk phase.^[^ ^29^ ^]^ Our measurements show that the surface‐supported complexes display a faster magnetization relaxation, considering the faster sweeping rate of the magnetic field used here. The stronger interaction of the Cp*ErCOT complexes results from the hybridization of the π orbitals of the standing‐up complexes directly with the metal, which breaks the symmetry of the ligand field and makes the complexes prone to vibronic coupling and dynamic charge transfer with the metal, all of which enhance the magnetization relaxation.^[^ ^19^, ^20^, ^21^, ^22^, ^23^, ^24^ ^]^ The X‐ray‐induced demagnetization has been estimated to give a minor contribution to the closing of the magnetic hysteresis, given the low photon flux and the molecular density, as compared to previous studies and K[Er(COT)_2_].^[^ ^26^, ^59^ ^]^

Contrarily, K[Er(COT)_2_] exhibits a strong net magnetic anisotropy due to the orientation of the molecular easy axes parallel to the substrate plane. The hysteresis opening proves clearly the slow relaxation of the magnetization, resembling the butterfly opening reported for the bulk phase of [K(18‐c‐6)][Er(COT)_2_]·2THF.^[^ ^33^, ^38^ ^]^ A direct comparison of the hysteresis loops is challenging because of different field sweep rates. However, following up on the literature on the bulk phase, we ascribe the temperature‐assisted quantum tunneling of magnetization process to be the dominant one, due to the similarities in the low field region. Yet, also here X‐ray‐induced demagnetization is expected to give a small contribution at low magnetic fields.

In the present study, the different hysteretic behavior of the two complexes is attributed to the orientation of the ligands’ π orbitals with respect to the substrate which allows or suppresses hybridization and thus favors or suppresses vibrational coupling to the Ag(100) substrate. The direct interaction of the π orbitals of standing‐up Cp*ErCOT with the substrate induces faster magnetization relaxation. On the other hand, because of the preferentially lying‐down orientation of K[Er(COT)_2_], the π orbitals are parallel to the surface plane and thus weakly interacting with the substrate. Importantly, due to this mechanism, the orientation of the complexes has an enormous impact on the magnetic relaxation properties.

## 3 Conclusion

The present study shows how structurally similar lanthanide organometallic SIMs based on π‐conjugated ring‐shaped ligands can exhibit completely different self‐assemblies on metal surfaces, due to different intermolecular interactions, as well as different degrees of interaction with the Ag(100) substrate. Cp*ErCOT forms compact rows of alternating standing‐up and lying‐down complexes along the [010] and [001] directions of the substrate. K[Er(COT)_2_] arranges in highly ordered domains with complexes purely in the lying‐down conformation, stabilized by the coordinated K^+^ ion. Polarized XAS indicates that the net magnetic anisotropy is strong for surface‐adsorbed K[Er(COT)_2_] and weak for Cp*ErCOT. Also, Cp*ErCOT exhibits weak hysteresis opening both in‐plane and out‐of‐plane because of the stronger interaction of the standing‐up complexes with the substrate through their conjugated π orbitals. Contrarily, the hysteresis opening of K[Er(COT)_2_] is large, suggesting a minor influence of the substrate on the magnetic relaxation properties. This is linked to the weak interaction of the π‐conjugated ligand orbitals with the substrate, given their orientation parallel to the substrate plane. This suggests that the π electron cloud directly interacting with the metallic surface results in an increased magnetization relaxation rate, possibly through enhanced vibronic molecule−substrate coupling, opposite to the case with the π orbitals oriented parallel to the metal substrate. This result is of great importance for the design of future surface‐adsorbed molecular architectures aiming to achieve stable and addressable molecular magnets.

## 4 Experimental Section

4.1

4.1.1

##### Synthesis of SIMs

The Cp*ErCOT and [K(18‐c‐6)][Er(COT)_2_]·2THF complexes were synthesized as described in the literature.^[^ ^29^, ^33^ ^]^

##### Sample Preparation

The air‐sensitive complexes were handled in He gas environment of a glovebox (H_2_O and O_2_ concentrations < 1 ppm). The samples were obtained in the range between a sub‐ML and a few MLs by thermal sublimation of bulk crystallites on freshly prepared single‐crystal Ag(100) surface in ultrahigh vacuum (UHV). Polycrystalline powder of [K(18‐c‐6)][Er(COT)_2_]·2THF was used as a source to deposit the K[Er(COT)_2_] complex, after degassing the (18‐crown‐6) and THF molecules below the sublimation temperature of 360 °C used for the samples shown in the text. The molecular coverage of the samples was estimated by a quartz crystal microbalance, calibrated by STM. The extracted coverage was cross‐referenced with the adlayer thickness obtained from the attenuation of the Ag 3 d levels of the substrate measured by XPS. Further details are reported in the Supporting Information.

##### XPS

Survey scans were recorded by using monochromatic (Δ*E* = 0.1 eV) synchrotron light at 800 eV at the PEARL beamline at the SLS, Paul Scherrer Institute.^[^ ^60^ ^]^ A front‐end aperture of 3 × 4 mm^2^, an exit slit of 30 μm, and a dwell time of 0.5 s per step were used. Detailed scans were recorded with energy steps between 0.03 and 0.5 eV.

##### STM

Images were acquired at a temperature of 4.5 K using an Omicron LT‐STM and post‐processed with Gwyddion.^[^ ^61^ ^]^ Atomically resolved scans of the bare Ag(100) surface were acquired for reference.

##### XAS

The XAS, XLD, and XMCD spectra were acquired in total electron yield mode using on‐the‐fly scans at the EPFL/PSI X‐Treme beamline at the SLS.^[^ ^62^ ^]^ To avoid beam‐induced damage of the samples, a defocused beam and a reduced photon flux were employed by minimizing the exit slit opening. The average photon flux used to measure the *M*(*H*) curves of the Cp*ErCOT samples was 2 × 10^−2^ ph nm^−2^ s^−1^, while the maximum flux used for K[Er(COT)_2_] was 5 × 10^−2^ ph nm^−2^ s^−1^. The applied magnetic field was always collinear with the X‐ray beam. To obtain hysteresis loops, the two circular polarizations were recorded separately for the two sweep directions. The intensity difference between the absorption at the energy of maximum XMCD and the pre‐edge was evaluated while sweeping the magnetic field at a rate of 2 T min^−1^. The *M*(*H*) curves were rescaled to the total magnetic moments extracted by the sum rule analysis. XAS is defined as the sum of the two polarized absorption spectra (*μ*_V_ + *μ*_H_) or (*μ* ^+^ + *μ* ^−^). The XLD is defined as the difference *μ*_V_–*μ*_H_, while the XMCD as *μ*+–*μ* ^−^. The values expressed in Table 1 and 2 are given in % with respect to the most intense corresponding XAS peak.

##### XAS Simulations

Details of the simulations performed using the multiX software are reported in the Supporting Information.

## Conflict of Interest

The authors declare no conflict of interest.

## Author Contributions

V.R. data curation: lead; investigation: lead; validation: lead; visualization: lead; writing—original draft: lead; M.B. resources: equal; writing—review and editing: supporting; M.H. investigation: supporting; writing—review and editing: supporting; D.V. investigation: supporting; writing—review and editing: supporting; K.H. resources: supporting; writing—review and editing: supporting; N.D. investigation: supporting; writing—review and editing: supporting; B.D. investigation: supporting; software: lead; writing—review and editing: supporting; M.D.K. resources: supporting; writing—review and editing: supporting; M.M. resources: supporting; supervision: supporting; writing—review and editing: supporting; C.C. funding acquisition: supporting; resources: supporting; supervision: supporting; writing—review and editing: supporting; M.M. funding acquisition: supporting; resources: supporting; supervision: supporting; writing—review and editing: supporting; F.N. supervision: supporting; writing—review and editing: supporting; J.D. funding acquisition: lead; project administration: lead; supervision: lead; writing—review and editing: lead.

## Supporting information

Supplementary Material

## Data Availability

The data that support the findings of this study are available from the corresponding author upon reasonable request.
