# Supplementary material for: Orientation‐Driven Large Magnetic Hysteresis of Er(III) Cyclooctatetraenide‐Based Single‐Ion Magnets Adsorbed on Ag(100)
Source: Small Sci. 2024 Apr 24;4(8):2400115. doi: 10.1002/smsc.202400115 (PMC11934973; doi:10.1002/smsc.202400115)
Supplement: Supplementary file 1 — Supplementary Material [file SMSC-4-2400115-s001.pdf]

Supporting Information

Orientation-Driven Large Magnetic Hysteresis of Er(III) Cyclooctatetraenide-Based Single-Ion Magnets Adsorbed on Ag(100)

*Vladyslav Romankov, Moritz Bernhardt, Martin Heinrich, Diana Vaclavkova, Katie Harriman, Niéli Daffé, Bernard Delley, Maciej Damian Korzyński, Matthias Muntwiler, Christophe Copéret, Muralee Murugesu, Frithjof Nolting, Jan Dreiser**

Table of Contents

1. Sample preparation 2
2. Stoichiometric characterization of K[Er(COT)$_2$] 2
3. Additional STM images 6
4. MultiX simulations 7
5. Additional XAS, XLD and XMCD of multilayer and powder samples 10
6. Extracted magnetic moments from XMCD sum rules 12
7. Additional hysteresis loops 15
8. MultiX input file 16
9. References 17

1. Sample preparation

Polycrystalline powders of [K(18-c-6)][Er(COT)$_2$]·2THF and Cp*ErCOT were used as starting materials.[1,2] Quartz crucibles were filled with ~10 mg of powder and transferred to the preparation systems of the X-Treme[3] and PEARL[4] beamlines. Due to the high oxygen and moisture sensitivity, the materials were handled in the inert He environment of a glovebox. The degassing and sublimation were performed by using a commercial organic effusion cell (Kentax) at the X-Treme beamline and a custom-made multi-pocket evaporator at PEARL. The complexes were deposited on the (100) surface of an Ag single crystal freshly prepared by Ar^+ sputter-annealing cycles. The deposition rate was measured using a quartz crystal microbalance.

2. Stoichiometric characterization of K[Er(COT)$_2$]

The yellow-colored [K(18-c-6)][Er(COT)$_2$]·2THF powder was degassed up to 107°C, after which several control samples were prepared at increasingly higher crucible temperatures of up to 360°C. The shifts of the XPS core levels as a function of the sublimation temperature of the compound and the coverage/thickness of the sample adlayer are reported in **Figure S1**. The background removal was performed by subtraction of a Shirley function in the C 1s and K 2p spectra, as well as spectra of Er 4d level of higher coverage samples (7 and 8 MLs), which display a larger signal-to-noise ratio. In the Er 4d spectra of samples with lower coverage and in all O 1s spectra a straight line was subtracted. The main C 1s core level peaks as shown in Figure S1 were normalized to unity for a better comparison of the features and chemical shifts. The intensities of the other core level spectra are normalized by the same factors used to normalize the C 1s core levels.

When the complex is sublimed at a crucible temperature of 107°C (red curves), a strong presence of carbon at 286.3 eV and oxygen at 532.9 eV is detected, while erbium and potassium are essentially negligible. The peaks are attributed to the deposition of large amounts of (18-c-6) crown ether molecules on the surface, due to the typical binding energy of the oxygen-bound carbon in such molecules[5,6] and the absence of the carbon sp^2 peak that would appear in the presence of tetrahydrofuran (THF) and/or COT^{2-}.[7]

In the range from 200°C to 320°C (purple curves), we found the presence of C, Er and K. Together with a strong O 1s peak, the presence of these elements and two different carbon environments suggests that the sample has the same elements as the polycrystalline starting material of [K(18-c-6)][Er(COT)$_2$]·2THF. Indeed, the latter compound has 16 aromatic carbon

atoms and 4 carbon atoms of the 2 THF molecules not bound to oxygen, while there are 12 oxygen-bound carbons in the crown ether and 4 in the 2 THF molecules. The ~1 ML sample produced at this temperature shows a ratio of the two carbon peak areas of 1.36 (obtained from fits using Voigt functions), which is in good agreement with the ratio of 1.25 expected for the polycrystalline starting material.

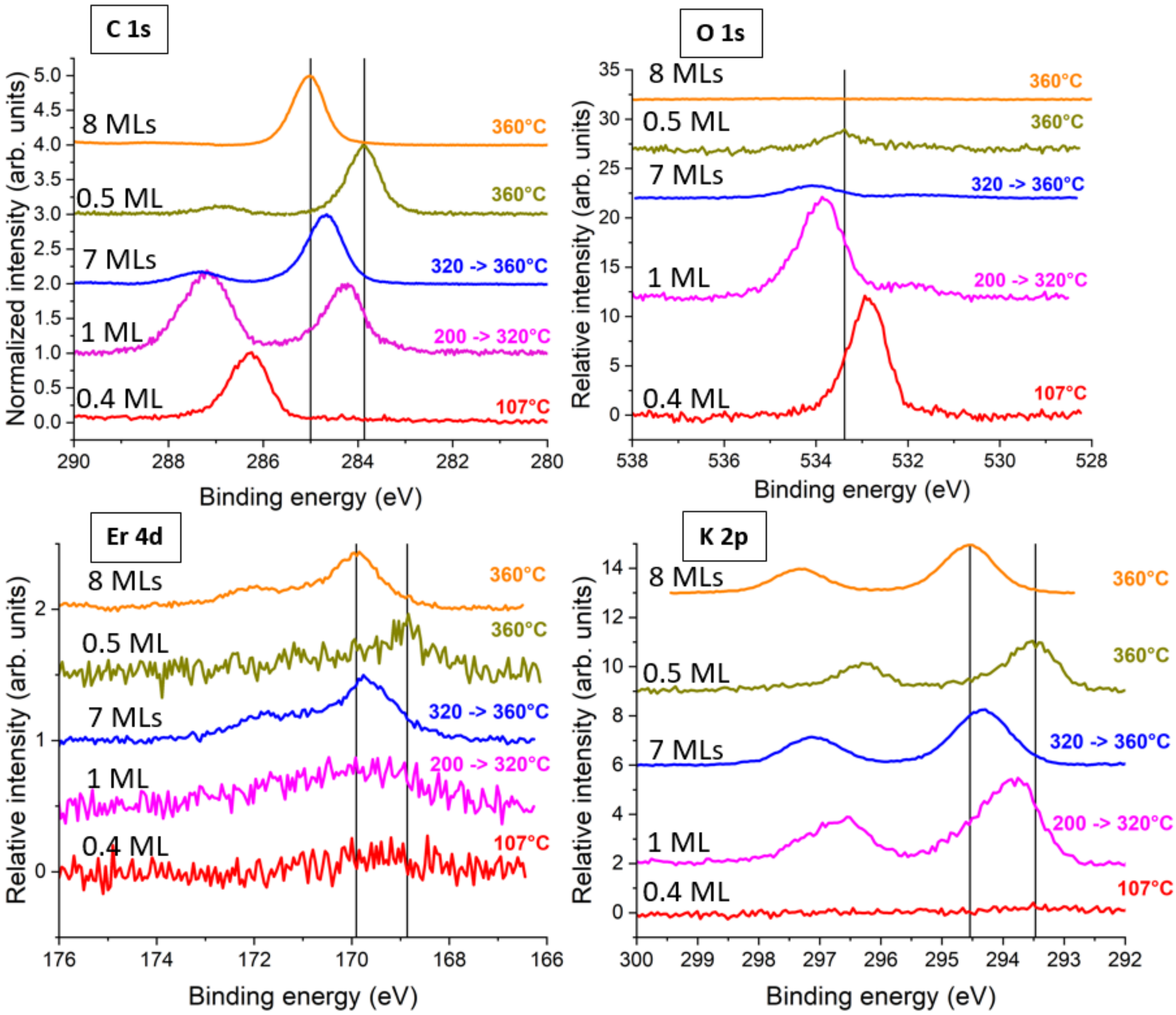

Figure S1. X-ray photoelectron spectra of C 1s, O 1s, Er 4d and K 2p core levels of K(18-c-6)[Er(COT)$_2$]·2THF deposited at different crucible temperatures on an Ag(100) substrate. While the C 1s peak is normalized to unity, the other edges are scaled by the same factors used for the C peak. The estimated coverage of the sample is indicated for each spectrum. The black vertical lines indicate the central position of the main core level peaks of the sample reported in the main text (dark yellow).

For crucible temperatures of 320-360°C (blue curves), the dominant type of carbon becomes that of the aromatic rings. The strong Er and K presence, and the weak C-O carbon (at higher binding energy) and oxygen signals suggest that the dominant species on the surface is

$K[Er(COT)_2]$. From the blue curve in the C 1s spectra it is possible to deduce that the ratio between the C-O and the sp^2 carbon peak areas is only 0.23. Assuming the crown ether to be the only contamination species, this roughly corresponds to a ratio of one crown ether per three $K[Er(COT)_2]$ complexes.

At a temperature of 360°C, which has been used to prepare the $K[Er(COT)_2]$ samples reported in the main text (dark yellow and orange curves), the presence of potassium, erbium, the (almost) negligible oxygen and mainly the aromatic carbon peak at the C 1s edge suggest that the main species deposited at this temperature is $K[Er(COT)_2]$. This simple analysis is confirmed by the stoichiometry analysis, as reported in the main text. The small C-O carbon signal indicates the presence of impurities on the surface. If all impurities are assumed to be crown ether molecules, the ratio of the C-O and sp^2 peaks shows that at most *one* crown ether molecule per *seven* $K[Er(COT)_2]$ complexes is present. In the presence of THF or other C and O containing contaminants, the ratio of crown ether vs $K[Er(COT)_2]$ would be even lower than 1:7. In the case of the multilayer sample (orange curves) the signals of the contaminants almost vanish. As compared to Er, the area ratios of C, O and K peaks of this sample amount to 20.5, ~0 and 1, which are close to the expected values for $K[Er(COT)_2]$ without crown ether. This can be understood by the chronological sequence of the experiments and the subsequent, increasing loss of the crown ether from the crucible as the 0.5 ML sample was prepared before the 8 ML one. The negligible traces of C-O carbon in the C 1s core level spectrum of the 8 ML sample and the absence of the O 1s peak confirm that the (18-c-6) crown ether and the THF molecules are already sublimed at temperatures lower than 360°C.

Moreover, the spectra shown in Figure S1 exhibit a thickness-dependent shift of all core level peaks. While the sub-monolayer samples have the lowest binding energy for all core levels, increasingly thicker molecular layers show a progressive shift of all core level peaks toward higher binding energy. The two boundaries, given by the ~0.5 ML (dark yellow) and the ~8 MLs (orange) coverages, have the core levels shifted on average by 1.05 eV with respect to one another, as indicated by the position of black vertical lines in Figure S1 (excluding oxygen). This can also be seen in **Figure S2**, where the relative shift of all peaks toward lower binding energy vs. estimated coverage is plotted. The shifts have been attributed to the vertical charge separation of the complex at the surface, which causes the formation of a built-in electric potential shifting the core levels toward lower binding energies. We assume that the K^+ ions adsorb closer to the substrate because of the charge compensation of the metal surface. Because of the smaller influence of the metal substrate in the multilayer sample the charge separation

effect becomes negligible. This concept is depicted in **Figure S3**, where the molecular orientation of the self-assembled complexes on Ag(100) is shown. Indeed, thickness-dependent test samples show a consistent shift of all core levels toward higher binding energies, in line with other polar materials reported in the literature.[8,9] Indeed, the samples with larger coverages tend to recover the position of the core levels, which are more aligned with the data reported for C 1s,[10–12] K 2p[13,14] and Er 4d.[15,16] In particular, the binding energy of the C 1s peak of the thickest test sample (8 MLs) coincides with the carbon peak of the π rings of the Cp*ErCOT(1 ML)/Ag(100) sample. Nevertheless, we do not exclude that the surface-induced screening effect or the charge accumulation of the thicker test samples can contribute to the shifts of the peak positions as well.

On the other side, small shifts of core levels to lower binding energies for sub-monolayer samples can also be attributed to the screening of the core hole performed by the image state electrons of metallic substrates. However, on metal surfaces such shifts are usually not greater than ~0.6 eV,[17] leading to a broadening of the peak, with a tail toward the lower binding energy side. In the case of fullerenes on Cu(111), the molecule-substrate interaction promoted shifts up to ~0.7 eV for the C 1s peak attributed to charger transfer[18], while on Au(100) the reported shift was -0.8 eV[19], similarly to other cases[17,20,21]. However, there is no clear indication of a charge transfer for K[Er(COT)$_2$]/Ag(100), since all the edges shift uniformly toward higher binding energies with increasing coverage.

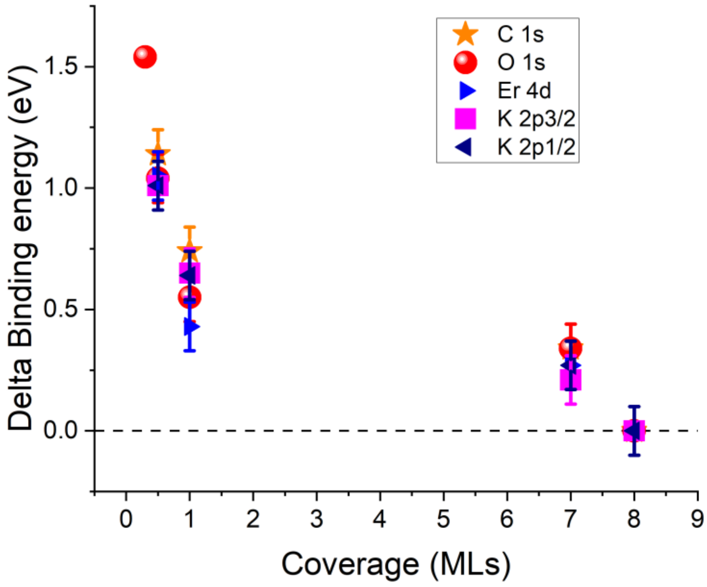

Figure S2. Thickness-dependent shift of the XPS core levels of K[Er(COT)$_2$] SMMs deposited on Ag(100), as explained in the text. The error bars are given by the FWHM of the different peaks.

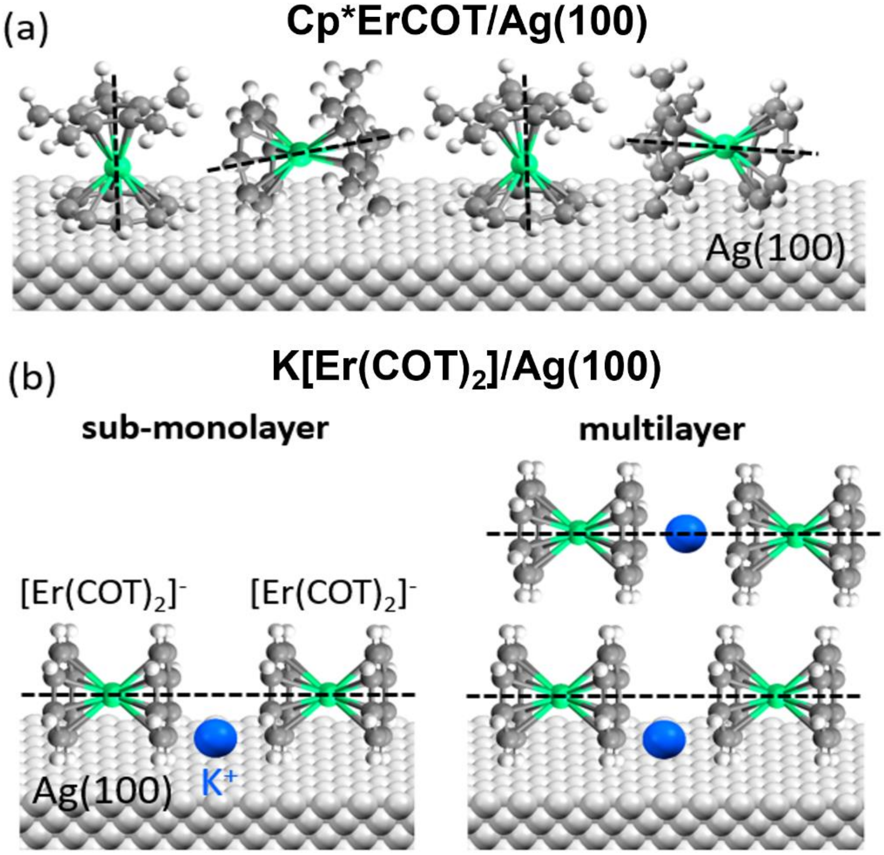

Figure S3. Model of (a) Cp*ErCOT/Ag(100) along the [-101] crystallographic direction and (b) $K[Er(COT)_2]$/Ag(100) along the axial direction as explained in the main text. The dashed lines represent the molecular axes of the complexes.

3. Additional STM images

In this section, we report two extra STM images of ~1 ML of Cp*ErCOT SMMs deposited on Ag(100). **Figure S4**a shows an area of 100 x 100 nm^2 acquired at 50 pA and 0.25 V. Highly oriented rows of molecular complex can be identified by an alternation of brighter and darker stripes in the diagonal direction of the Figure. The self-assembled complex forms multiple domains of rows with the same geometrical configuration, as explained in the main text. At the domain boundaries, the contrast of the rows is inverted, so that brighter rows become darker and vice-versa. Figure S4b shows an area of 500 x 500 nm^2 acquired at 50 pA and 0.25 V. Different terraces can be identified in the Figure, due to the step-like structure of the Ag substrate underneath. Line profile scans acquired across the terrace steps give a vertical step of

~0.25 ± 0.05 nm. Figure S4b also shows artifacts due to the mobility/dragging of the complex clusters under the STM tip.

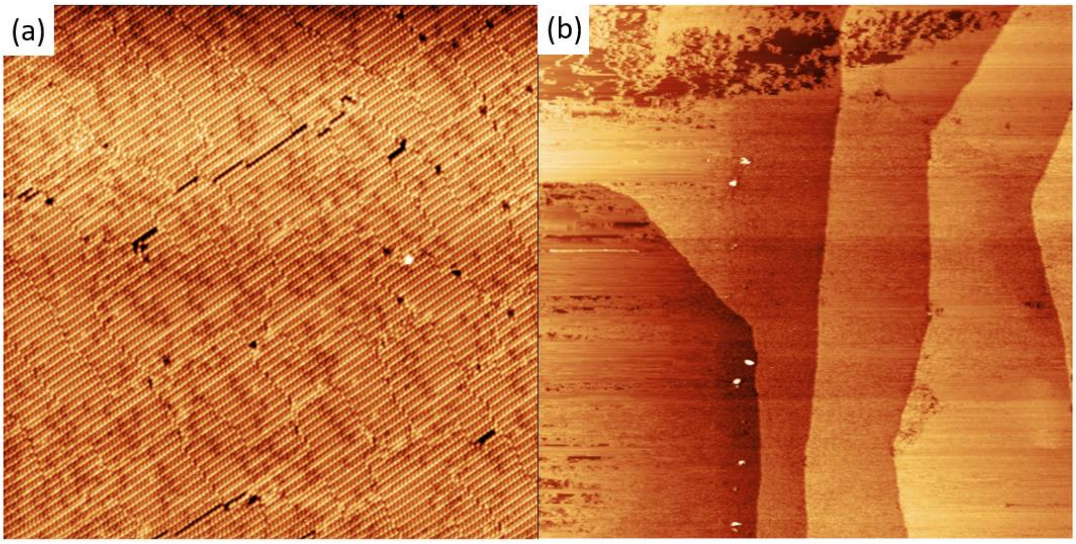

Figure S4. Constant-current STM images recorded at 4.5 K of Cp*ErCOT(~1 ML)/Ag(100). The imaging conditions are: (a) 100 x 100 nm^2, acquired at 50 pA and 0.25 V; (b) 500 x 500 nm^2, 50 pA and 0.25 V.

4. MultiX simulations

The multiX software[22] was used to simulate the XAS reported in the main text. In this method, the ligands surrounding the absorbing atom are taken into account by point charges. This simple yet powerful model is useful to understand the main features of the XAS, XLD and XMCD spectra and their dependence on the X-ray incidence angle and the applied magnetic field. An example input file can be found in Section 8 of this Supplementary Information.

The multiX approach relies significantly on first principles to calculate the details of the electronic structure of the central atom and its multiplet structure in a ligand field from very little input. The electron orbitals, which are needed to construct all determinant functions arising from the open shells in the ground and the excited states, are based on a local density approximation (LDA) self-consistent fully relativistic atomic calculation inside multiX. The LDA provides the gross excitation energy. It is only slightly corrected near the 1% level by a semiempirical threshold correction in the preset case. The spin-orbit splitting, setting the separation between $M_{4,5}$ edges, is scaled down by ~5% semiempirically. The electron-electron

interaction is scaled down by 15% from the bare orbital-based result. These two scalings are very typical.

The environment of the rare earth atom has a great influence on the details of the atomic ground state. For the present calculations, a simple model with only 2 x 8 carbon atomic positions is used. In principle, the delocalized electron cloud of the π orbitals of the ligand rings and their almost parallel planes can be parameterized as an effective ligand field of $C_{\infty v}$ symmetry, acting on the central Er^{3+} ion.[23] For this reason, in our model we assume that the effective point charges perceived by the Er^{3+} ions of the two complexes are very similar due to the sandwich structure of the systems and, although the coordination environment of the two compounds is different, the effective charge can be parameterized similarly. Hence the simulations are based on a structure with an enforced D_{8h} symmetry, which is realized by positioning accordingly the carbon atoms of the first coordination shell with the average Er-C bond distance taken from literature.[1,24] A single value of carbon point charge as a fit parameter for the crystal field/ ligand field is used as an actual fit parameter. The precise value of this fit parameter has a significant influence on the magnetic properties and is crucially defined by the XLD and XMCD measurements. The nominal positions are reported in **Table S1**. To keep the model simple and the number of parameters low, we manually changed the point charge parameter of the carbon atoms and the fraction of complexes oriented with the molecular axis in-plane or out-of-plane as compared to the substrate plane for both compounds. Furthermore, to match the experimental spectra, the Coulomb and spin-orbit interactions were scaled to 85% and 95% of the computed values, respectively. A core-hole lifetime broadening of 0.45 eV to 1.45 eV in the span of 1398 eV to 1440 eV was used to simulate the peak widths at the $M_{4,5}$-edges. The ligand field was scaled by a multiplication factor of 1.182.

Table S1. Atomic positions and charges used to generate the ligand field of a standing-up generic molecule in the D_{8h} symmetry implemented in the multiX code. The positions were extracted from ref. [1].

Atom	x (Å)	y (Å)	z (Å)	q_C (e)
C	0	1.831	1.912	0.25
C	1.2947	1.2947	1.912	0.25
C	1.831	0	1.912	0.25
C	1.2947	-1.2947	1.912	0.25
C	0	-1.831	1.912	0.25
C	-1.2947	-1.2947	1.912	0.25

C	-1.831	0	1.912	0.25
C	-1.2947	1.2947	1.912	0.25
C	0	1.831	-1.912	0.25
C	1.2947	1.2947	-1.912	0.25
C	1.831	0	-1.912	0.25
C	1.2947	-1.2947	-1.912	0.25
C	0	-1.831	-1.912	0.25
C	-1.2947	-1.2947	-1.912	0.25
C	-1.831	0	-1.912	0.25
C	-1.2947	1.2947	-1.912	0.25

All simulated spectra are based on linear combinations of three configurations of the model complex giving rise to different absorption spectra: the standing-up configuration, with the axis normal to the surface plane and the two lying-down configurations, with the axis rotated by 90° around the x- and y-axis. Since the grazing incidence was imposed by a tilt angle of 60° with respect to the y-axis, the in-plane molecules were simulated by the average of the spectra of the two lying-down configurations, oriented with the axis of the complex parallel to the x and y reference axes. The experimental conditions, i.e., the magnetic field and temperature, were considered: The magnetic field was simulated parallel to the X-ray beam direction. Its value was 50 mT and 6.8 T for the linearly and circularly polarized XAS, respectively.

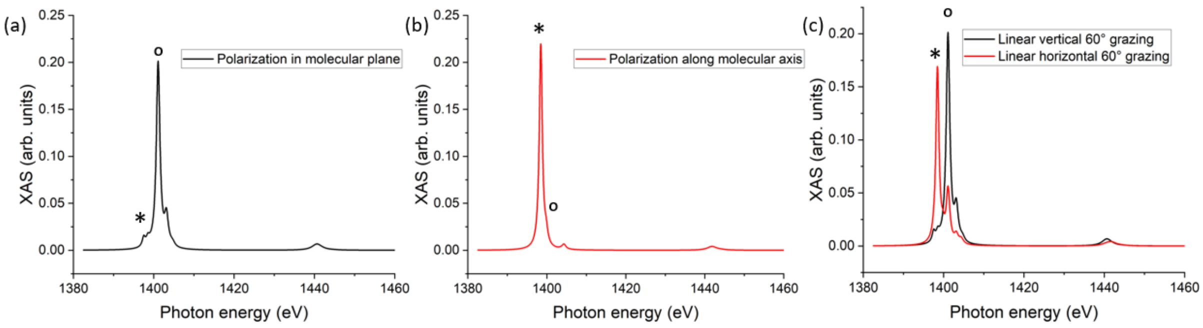

Figure S5. MultiX-simulated XAS at the Er $M_{4,5}$-edges as explained in the text. The spectra are shown for the molecular axis (a) perpendicular and (b) parallel to the molecular axis of the model system. (c) A spectrum obtained by using two perpendicular polarization vectors oriented at a grazing angle of 60° with respect to the molecular axis.

A model calculation for the case of linearly polarized X-rays is reported in **Figure S5**. When the polarization vector is oriented in the plane of the simulated COT^{2-} ligands, the absorption

spectra show a strong peak around 1401 eV ("°" feature) and negligible intensity at 1398 eV ("*" feature), as visible in Figure S5a. On the contrary, when the polarization vector is oriented along the main molecular axis the weight of the features changes (*cf*. Figure S5b). In the case of an incidence angle of 60° with respect to the molecular axis, a spectrum similar to the one in Figure S5c is obtained. A full multiX documentation can be found at the link: http://multiplets.web.psi.ch/

5. Additional XAS, XLD and XMCD of multilayer and powder samples

Figure S6 reports the XAS and XLD spectra of 2 MLs and 4 MLs K[Er(COT)$_2$]/Ag(100) and Cp*ErCOT/Ag(100), respectively, while **Figures S7** and **S8** show the circularly polarized and the XMCD spectra. The XMCD values are reported together with the subML samples in **Table 2** of the main text, for direct comparison. **Figure S9** displays the XAS and XMCD spectra of polycrystalline powder of [K(18-c-6)][Er(COT)$_2$]·2THF.

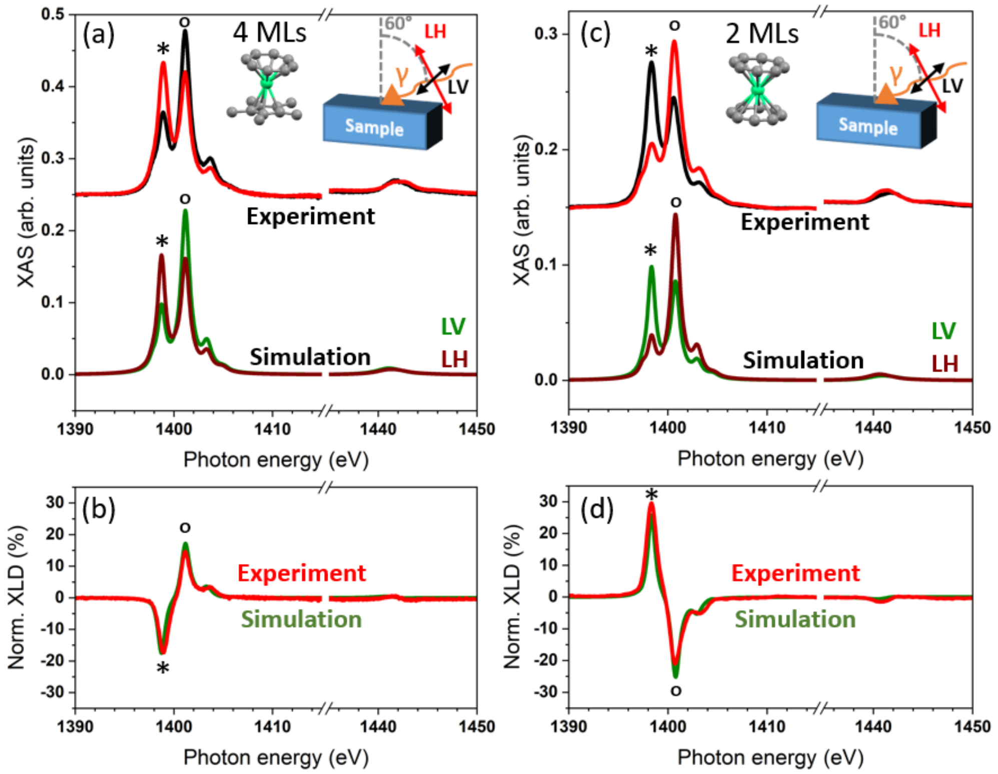

Figure S6. Linearly polarized XAS and XLD at the Er $M_{4,5}$-edges measured at 3 K at a grazing angle of 60° from the surface normal direction and multiX-simulated spectra of (a,b) Cp*ErCOT(4 ML)/Ag(100) and (c,d) K[Er(COT)$_2$](2 ML)/Ag(100).

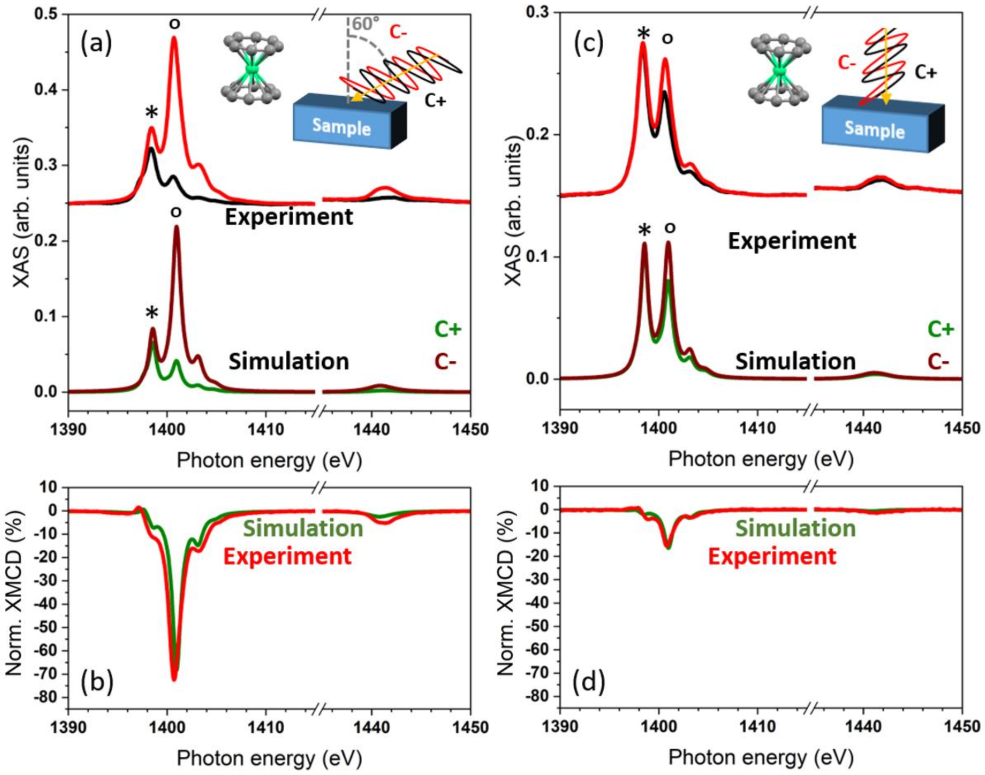

Figure S7. Circularly polarized XAS, XMCD and multiX-simulated spectra at the Er $M_{4,5}$-edges recorded in (a,b) grazing and (c,d) normal incidence of coverage of K[Er(COT)$_2$](2 ML)/Ag(100). T = 3 K and B = 6.8 T.

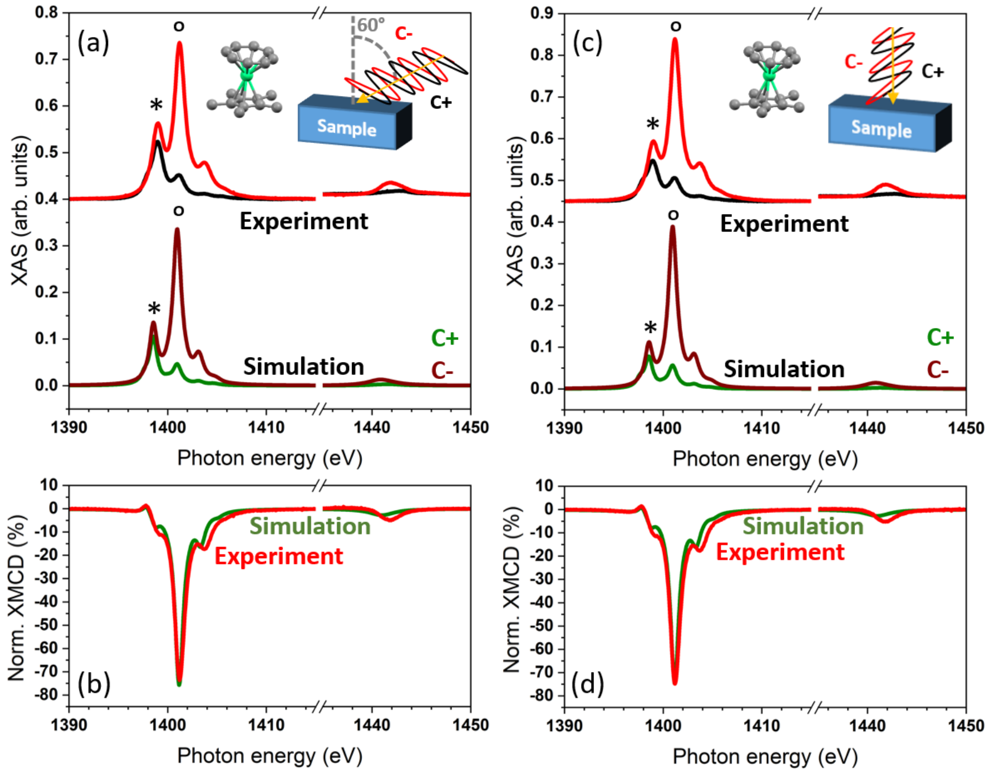

Figure S8. Circularly polarized XAS, XMCD recorded at the Er $M_{4,5}$-edges on Cp*ErCOT(4 ML)/Ag(100) and multiX-simulated spectra in (a,b) grazing and (c,d) normal incidence. T = 3 K and B = 6.8 T.

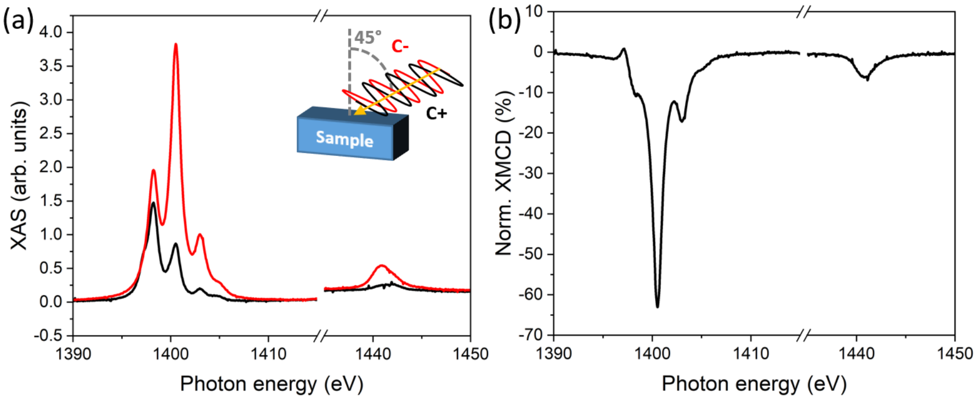

Figure S9. (a) Circularly polarized XAS and (b) XMCD recorded at the Er $M_{4,5}$-edges on a polycrystalline powder sample of [K(18-c-6)][Er(COT)$_2$]·2THF. T = 3 K and B = 6.8 T. The powder was pressed in indium foil, which was attached to the sample holder. The preparation and the transfer to the vacuum system of the X-Treme beam line were performed under helium gas atmosphere.

6. Extracted magnetic moments from XMCD sum rules

Sum rule analysis[25,26] of the Er $M_{4,5}$ XMCD spectra was performed to obtain the element-specific magnetic moment of erbium. To compare the experimental magnetic moment values to the theoretical ones, sum rule analysis was also performed on the multiX-simulated spectra. The results are reported in **Tables S2** and **S3**. The $<L_Z>$ and $<S_{Zeff}>$ values extracted from the spectra in Figures 4 and 5 are similar to the ones extracted from the corresponding multiX simulations. In order to determine the $<S_Z>$ values from $<S_{Zeff}>$ we assumed that the magnetic dipole moment $<Tz>$ is proportional to $<S_{Zeff}>$, so when the molecular easy-axis is parallel to the field and the incoming photons the magnitude has the maximum value of the free Er^{3+} ion $<Tz>$.[27] For other geometries, we scaled the value by the same fraction factor that $<S_{Zeff}>$ scales with respect to its maximum value.

For Cp*ErCOT(0.5 ML)/Ag(100) the total magnetic moment in normal incidence is about half of the expected value for the pristine molecule since only half of the molecules have the magnetic easy-axis aligned with the field. The values in normal incidence are well reproduced by the simulations, but the experimental moments in grazing incidence are smaller than the values obtained by multiX. While the magnetic moment in grazing incidence is expected to be smaller than in normal incidence because of the angle-dependent projection along the beam direction, an anisotropic ordering of the molecules in the substrate plane can reduce the easy-axis contribution in the XMCD spectrum. On the other side, defects in the form of standing-up molecules, as seen in the STM images, can also contribute to a larger out-of-

plane magnetization. Since changing the point charges of the carbon atoms or the standing-up vs. lying-down ratio of the molecules worsens the agreement of the simulations with the experimental spectra, we assume other parameters are relevant for the simulation (for example the anisotropic orientation of the lying-down molecules).

For $K[Er(COT)_2]$(0.5 ML)/Ag(100) the total magnetic moments of 4.5 ± 0.8 μ_B in grazing against the 1.9 ± 0.8 μ_B in normal incidence fits the anisotropy of the net magnetization resulting from the lying-down geometry. The experimental and simulated values are in excellent agreement, corroborating the strength of the point-charge model based on the D_{8h} symmetry with the point charge of the carbon atoms of q_C = 0.25 e and a fraction of 13% of standing-up complexes.

The sum rule results of the multilayer samples show an excellent agreement between the experimental values and the ones extracted from the simulated spectra, as shown in Table S3. At low temperature and high magnetic field, the total Er magnetic moment of the powder sample of the starting material $[K(18\text{-}c\text{-}6)][Er(COT)_2]\cdot 2THF$ reported in Figure S9 and Table S4 is about half of the value expected for free, isotropic Er^{3+} ions (9 μ_B). The reduction of ~0.5 compared to the free ion value is consistent with the randomly oriented sample of strongly anisotropic complexes.[28]

Table S2. Orbital, spin and total magnetic moment values extracted from the experimental and simulated spectra of samples with 0.5 ML coverages (Figures 4 and 5). The units are [μ_B].

Cp*ErCOT	Normal		Grazing	
	Experiment	MultiX	Experiment	MultiX
m_L	3.2 ± 0.8	3.3	2.4 ± 0.6	3.2
m_S	0.9 ± 0.2	0.8	0.4 ± 0.1	0.8
m_B	5.0 ± 1.0	4.9	3.3 ± 0.7	4.7
$K[Er(COT)_2]$	Normal		Grazing	
	Experiment	MultiX	Experiment	MultiX
m_L	1.2 ± 0.5	1.2	3.1 ± 0.7	3.4
m_S	0.4 ± 0.3	0.3	0.7 ± 0.1	0.8
m_B	1.9 ± 0.8	1.8	4.5 ± 0.8	5.0

Table S3. Orbital, spin and total magnetic moment values extracted from the experimental and simulated spectra of multilayer samples reported in Figures S7 and S8. The units are [μ_B].

4 ML Cp*ErCOT	Normal		Grazing	
	Experiment	MultiX	Experiment	MultiX
m_L	3.3 ± 0.8	3.3	2.9 ± 0.7	3.2
m_S	0.8 ± 0.2	0.8	0.7 ± 0.1	0.8
m_{tot}	4.9 ± 0.9	4.9	4.3 ± 0.9	4.7
2 ML $K[Er(COT)_2]$	Normal		Grazing	
	Experiment	MultiX	Experiment	MultiX
m_L	0.5 ± 0.2	0.5	3.4 ± 0.5	3.0
m_S	0.11 ± 0.03	0.1	0.7 ± 0.1	0.7
m_{tot}	0.7 ± 0.3	0.8	4.9 ± 0.6	4.4

Table S4. Orbital, spin and total magnetic moment values extracted from the experimental XAS/XMCD of the polycrystalline powder of $[K(18\text{-}c\text{-}6)][Er(COT)_2]\cdot 2THF$ reported in Figure S9. The units are [μ_B].

	Magnetic Moment
m_L	2.9 ± 0.3
m_S	0.7 ± 0.1
m_{tot}	4.3 ± 0.5

7. Additional hysteresis loops

Figures S10 and **S11** reports the XMCD-detected magnetic hysteresis loops of Cp*ErCOT/Ag(100) and K[Er(COT)$_2$/Ag(100)], respectively. The spectra are obtained at different coverages and are normalized to the value of saturation of the magnetization. The saturation values of the lowest and largest coverages are reported in Tables S2 and S3.

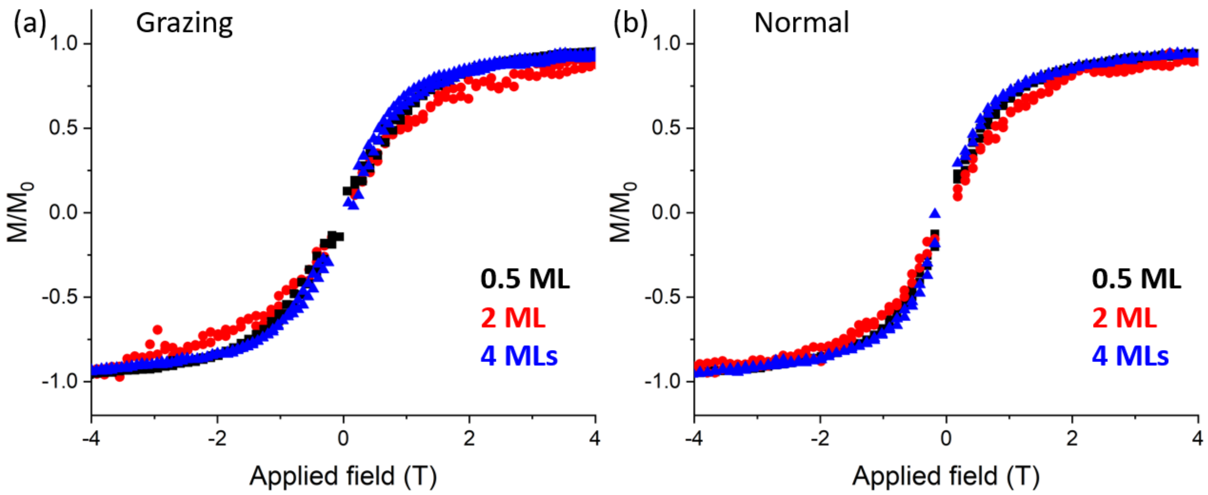

Figure S10. XMCD-detected magnetic hysteresis loops of Cp*ErCOT/Ag(100) recorded at 3 K and a rate of 2 T/min on samples with 0.5, 2 and 4 MLs coverage. Normal stands for out-of-plane direction, while grazing (60°) is mostly in-plane.

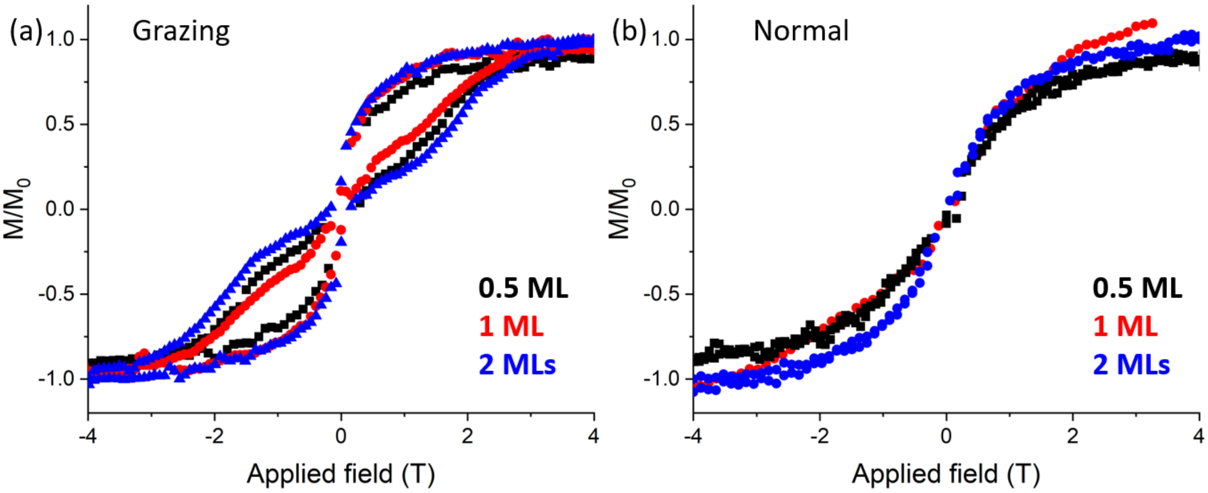

Figure S11. XMCD-detected magnetic hysteresis loops of K[Er(COT)$_2$]/Ag(100) recorded at 3 K and a rate of 2 T/min on samples with 0.5, 1 and 2 MLs coverage. Normal stands for out-of-plane direction, while grazing (60°) is mostly in-plane.

8. MultiX input file

```
new XMCD
atom Er
ground_state 3d10 4f12

scaler_coulomb 0.85
scaler_so_coupling 0.95
scaler_xtal_field 1.182
threshold_corr 22.5

core_hole_broad 0.50
deltag1         1.00
wmingl 1398.0
wmaxg1 1440.0
temperature 3 #used for the population of the sub-states

#the beam direction is in the xyz frame, where xy is the substrate
#plane (y pointing "up" in the experimental configuration) and z is
#out-of-plane direction

bfield 6.8
beam_in    -0.886 -0.5 0   #beam direction in grazing 60 deg
bfield_dir -0.886 -0.5 0 #magnetic field direction in grazing 60 deg

begin_xtal
1.000000 [Angstroems] x y z q radius
 -1.8310   1.9120    0       0.2500
 -1.2947   1.9120  -1.2947   0.2500
   0       1.9120  -1.8310   0.2500
  1.2947   1.9120  -1.2947   0.2500
  1.8310   1.9120    0       0.2500
  1.2947   1.9120   1.2947   0.2500
   0       1.9120   1.8310   0.2500
 -1.2947   1.9120   1.2947   0.2500
 -1.8310  -1.9120    0       0.2500
 -1.2947  -1.9120  -1.2947   0.2500
   0      -1.9120  -1.8310   0.2500
  1.2947  -1.9120  -1.2947   0.2500
  1.8310  -1.9120    0       0.2500
  1.2947  -1.9120   1.2947   0.2500
   0      -1.9120   1.8310   0.2500
 -1.2947  -1.9120   1.2947   0.2500
end_xtal

spect_emin  1390
spect_emax  1450

emid_xmcd   1420
QNprint     14
GSanalyze
```

9. References

[1] K. R. Meihaus, J. R. Long, *J. Am. Chem. Soc.* **2013**, *135*, 17952.

[2] J. J. Le Roy, M. Jeletic, S. I. Gorelsky, I. Korobkov, L. Ungur, L. F. Chibotaru, M. Murugesu, *J. Am. Chem. Soc.* **2013**, *135*, 3502.

[3] C. Piamonteze, U. Flechsig, S. Rusponi, J. Dreiser, J. Heidler, M. Schmidt, R. Wetter, M. Calvi, T. Schmidt, H. Pruchova, J. Krempasky, C. Quitmann, H. Brune, F. Nolting, *J. Synchrotron Radiat.* **2012**, *19*, 661.

[4] M. Muntwiler, J. Zhang, R. Stania, F. Matsui, P. Oberta, U. Flechsig, L. Patthey, C. Quitmann, T. Glatzel, R. Widmer, E. Meyer, T. A. Jung, P. Aebi, R. Fasel, T. Greber, *J. Synchrotron Radiat.* **2017**, *24*, 354.

[5] S. Tas, O. Kaynan, E. Ozden-Yenigun, K. Nijmeijer, *RSC Adv.* **2016**, *6*, 3608.

[6] E. E. Johnston, J. D. Bryers, B. D. Ratner, *Langmuir* **2005**, *21*, 870.

[7] G. R. Zhuang, K. Wang, Y. Chen, P. N. Ross, *J. Vac. Sci. Technol. A Vacuum, Surfaces, Film.* **1998**, *16*, 3041.

[8] S. A. Chambers, L. Qiao, T. C. Droubay, T. C. Kaspar, B. W. Arey, P. V. Sushko, *Phys. Rev. Lett.* **2011**, *107*, 206802.

[9] T. C. Taucher, I. Hehn, O. T. Hofmann, M. Zharnikov, E. Zojer, *J. Phys. Chem. C* **2016**, *120*, 3428.

[10] C. J. Groenenboom, G. Sawatzky, H. J. de Liefde Meijer, F. Jellinek, *J. Organomet. Chem.* **1974**, *76*, C4.

[11] M. Briganti, G. Serrano, L. Poggini, A. L. Sorrentino, B. Cortigiani, L. C. de Camargo, J. F. Soares, A. Motta, A. Caneschi, M. Mannini, F. Totti, R. Sessoli, *Nano Lett.* **2022**, *22*, 8626.

[12] C. M. Woodbridge, D. L. Pugmire, R. C. Johnson, N. M. Boag, M. A. Langell, *J. Phys. Chem. B* **2000**, *104*, 3085.

[13] A. Cano, I. Monroy, M. Ávila, D. Velasco-Arias, J. Rodríguez-Hernández, E. Reguera, *New J. Chem.* **2019**, *43*, 18384.

[14] M. Ayyoob, M. S. Hegde, *Surf. Sci.* **1983**, *133*, 516.

[15] C. Mao, W. Li, F. Wu, Y. Dou, L. Fang, H. Ruan, C. Kong, *J. Mater. Sci. Mater. Electron.* **2015**, *26*, 8732.

[16] N. G. Kalugin, A. J. Roy, K. Artyushkova, A. Serov, *Nanotechnology* **2017**, *28*, 195603.

[17] S. J. Chase, W. S. Bacsa, M. G. Mitch, L. J. Pilione, J. S. Lannin, *Phys. Rev. B* **1992**,

46, 7873.

[18] K. D. Tsuei, J. Y. Yuh, *Phys. Rev. B - Condens. Matter Mater. Phys.* **1997**, *56*, 15412.

[19] A. V. Akimov, C. Williams, A. B. Kolomeisky, *J. Phys. Chem. C* **2012**, *116*, 13816.

[20] B. Hoogenboom, R. Hesper, L. Tjeng, G. Sawatzky, *Phys. Rev. B - Condens. Matter Mater. Phys.* **1998**, *57*, 11939.

[21] M. R. C. Hunt, S. Modesti, P. Rudolf, R. E. Palmer, *Phys. Rev. B* **1995**, *51*, 10039.

[22] A. Uldry, F. Vernay, B. Delley, *Phys. Rev. B* **2012**, *85*, 125133.

[23] S.-D. Jiang, B.-W. Wang, H.-L. Sun, Z.-M. Wang, S. Gao, *J. Am. Chem. Soc.* **2011**, *133*, 4730.

[24] L. Ungur, J. J. Leroy, I. Korobkov, M. Murugesu, L. F. Chibotaru, *Angew. Chemie - Int. Ed.* **2014**, *53*, 4413.

[25] B. T. Thole, P. Carra, F. Sette, G. van der Laan, *Phys. Rev. Lett.* **1992**, *68*, 1943.

[26] P. Carra, B. T. Thole, M. Altarelli, X. Wang, *Phys. Rev. Lett.* **1993**, *70*, 694.

[27] Y. Teramura, A. Tanaka, T. Jo, *J. Phys. Soc. Japan* **1996**, *65*, 1053.

[28] J. Dreiser, K. S. Pedersen, C. Piamonteze, S. Rusponi, Z. Salman, M. E. Ali, M. Schau-Magnussen, C. A. Thuesen, S. Piligkos, H. Weihe, H. Mutka, O. Waldmann, P. Oppeneer, J. Bendix, F. Nolting, H. Brune, *Chem. Sci.* **2012**, *3*, 1024.
